# Supplementary figures and images for: Convergent evolution of H4K16ac-mediated dosage compensation in the ZW species Artemia franciscana
Source: PLoS Genet. 2025 Oct 9;21(10):e1011895. doi: 10.1371/journal.pgen.1011895 (PMC12527168; doi:10.1371/journal.pgen.1011895)

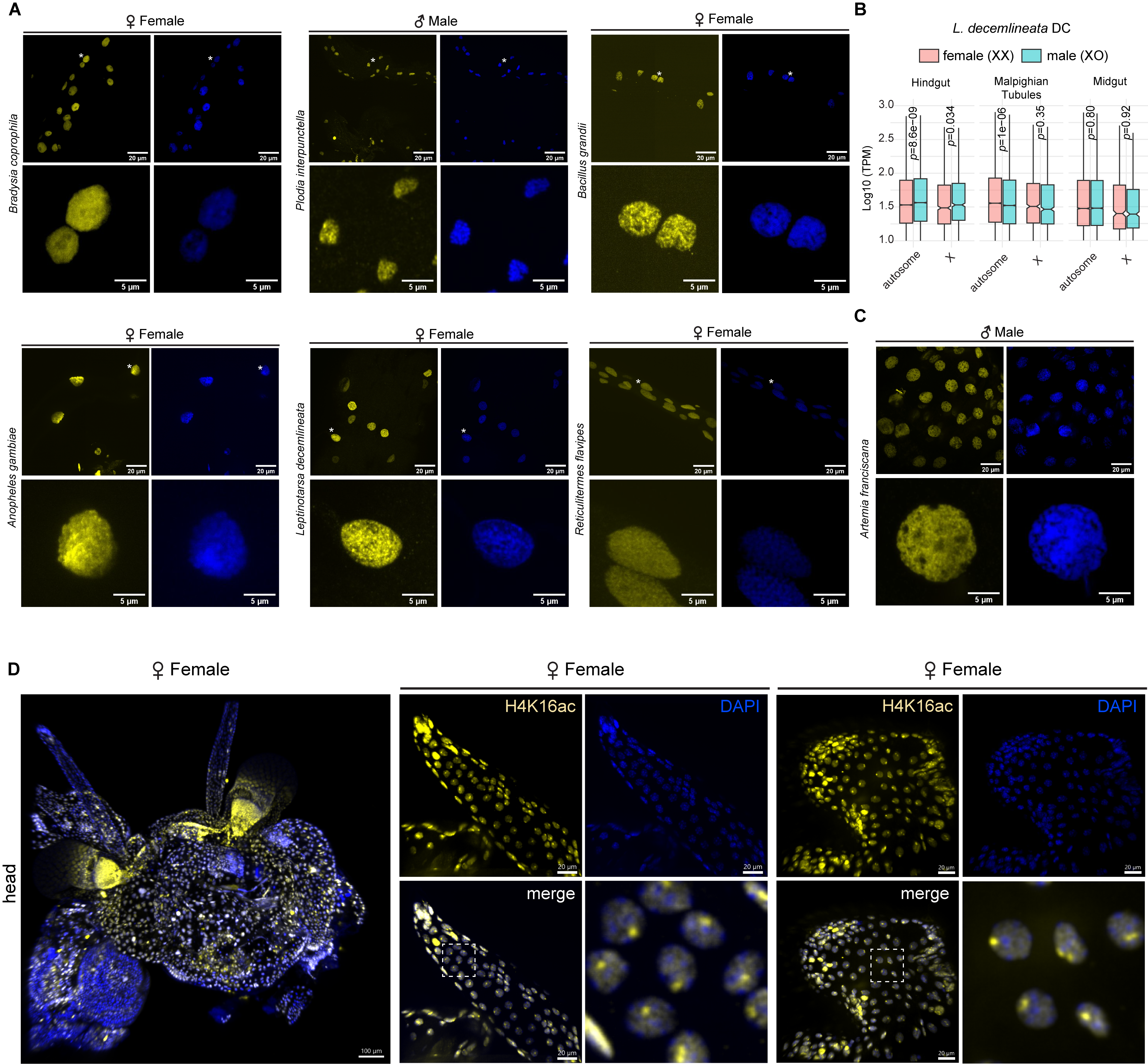

Supplement: S1 Fig — Immunofluorescence staining of H4K16ac in the indicated species with H4K16ac in yellow and DAPI in blue. All images represent maximum intensity projections of a Z-stack. (A) Homogametic sex of indicated species; the heterogametic sex that was stained/acquired as part of the same experiments are presented in Fig 1C. (B) Boxplot showing the L. decemlineata DC status from RNA-seq [86] in different tissues by comparing the overall expression in log10(TPM) on X and autosomes in males and females, respectively. Genes with <10 TPM were considered not expressed and thus removed from the analysis. p-values were calculated using a two-sided Wilcoxon rank-sum test. (C) Male A. franciscana epithelial cells of the abdomen. (D) Head of female A. franciscana, showing an overview (left), antennae (middle), and eyestalk (right). The areas highlighted with dashed squares are magnified in the bottom right panel of each 4-square assembly. (TIF) [file pgen.1011895.s001.tif]

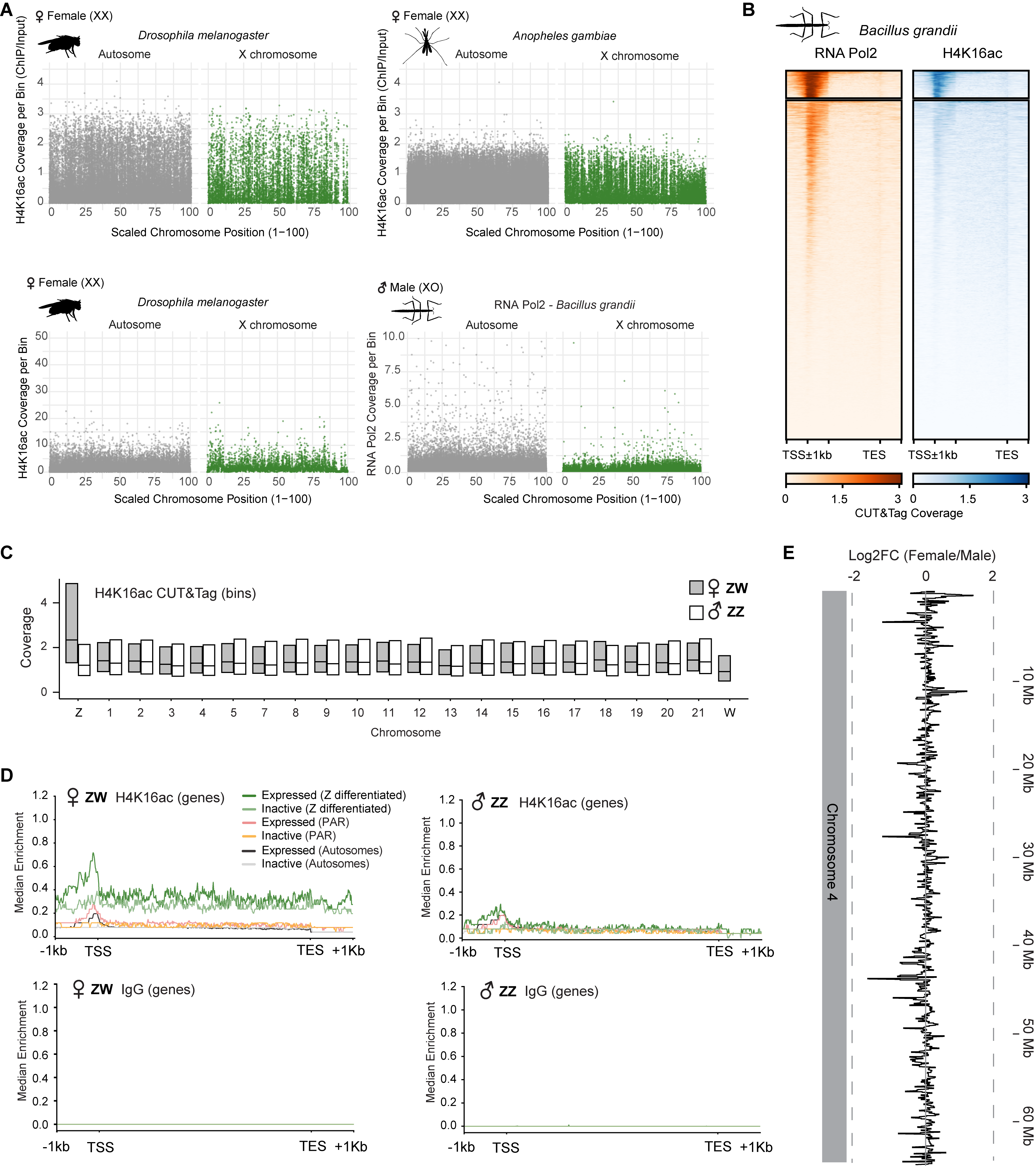

Supplement: S2 Fig — (A) Dot plots showing normalized coverage of H4K16ac (CUT&Tag or ChIP-seq, depending on species) in females. Shown is one representative replicate, with chromosomes segmented into 5 kb bins. The x-axis represents the relative position of each bin along the chromosome, scaled from 1 (chromosome start) to 100 (chromosome end). Similarly, RNA polymerase II coverage is shown for Bacillus grandii males (X0). Pictograms are from phylopic.org. (B) Heatmaps of normalized CUT&Tag coverage in Bacillus grandii for RNA polymerase II (left) and H4K16ac (right). Signals are plotted across gene bodies from transcription start site (TSS) to transcription end site (TES) with ±1 kb flanking regions. Pictograms are from phylopic.org. (C) Box plots showing the normalized H4K16ac CUT&Tag coverage of a single representative replicate for each chromosome, segmented into 10 kb bins, in females (grey) and males (white). The experiment was conducted from n = 3 biological replicates of each sex. (D) Normalized CUT&Tag enrichment for H4K16ac in comparison with IgG control in one representative female (left) and male (right) sample, shown as a metaplot depicting median enrichment across different gene groups. Gene bodies are scaled to 5 kb between the transcription start site (TSS) and transcription end site (TES), with an additional 1 kb extension upstream and downstream for visualization. Genes were classified as expressed based on a cutoff of TPM ≥ 10. H4K16ac CUT&Tag was independently conducted from n=3 biological replicates with similar results (S1 Data for details). The experiment shown here was conducted from frozen Artemia tissue. An independent experiment with freshly processed tissue is presented in Fig 3. Both experiments yield similar conclusions, with slightly improved signal-to-noise observed in the data from fresh tissue. (E) log2FC of H4K16ac levels between females and males across chromosome 4 (chosen as one representative autosomal example). (TIF) [file pgen.1011895.s002.tif]

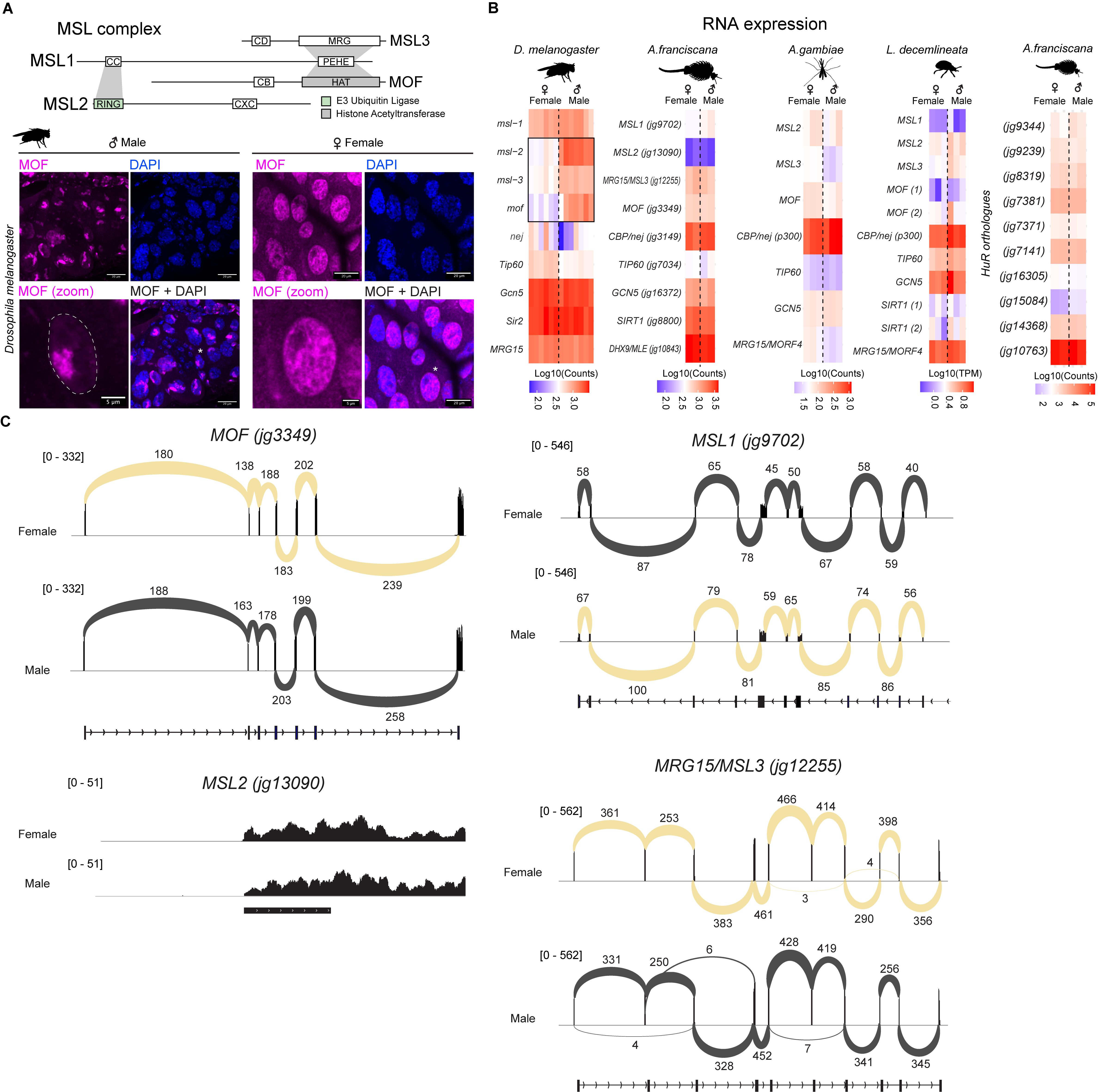

Supplement: S3 Fig — (A) Schematic representation of MSL complex architecture (top) and immunofluorescence staining of MOF (purple) and DAPI (blue) in male and female D.melanogaster. (B) Heatmap showing the normalized gene expression of MSL complex members as well as selected HAT and HDAC genes for D. melanogaster [57], A. franciscana [14], A. gambiae [23], and L. decemlineata [86]. An additional heatmap displays expression of HuR orthologs for A. franciscana [14]. Rows represent biological replicates and columns the different genes, respectively. Pictograms are from phylopic.org. (C) Sashimi plots showing the splicing patterns of MSL complex members in adult A. franciscana individuals of both females and males, generated using IGV. The y-axis represents the read coverage from the BAM file (not normalized), and the arcs below indicate the reads that span the exon-exon junctions as lines. (TIF) [file pgen.1011895.s003.tif]

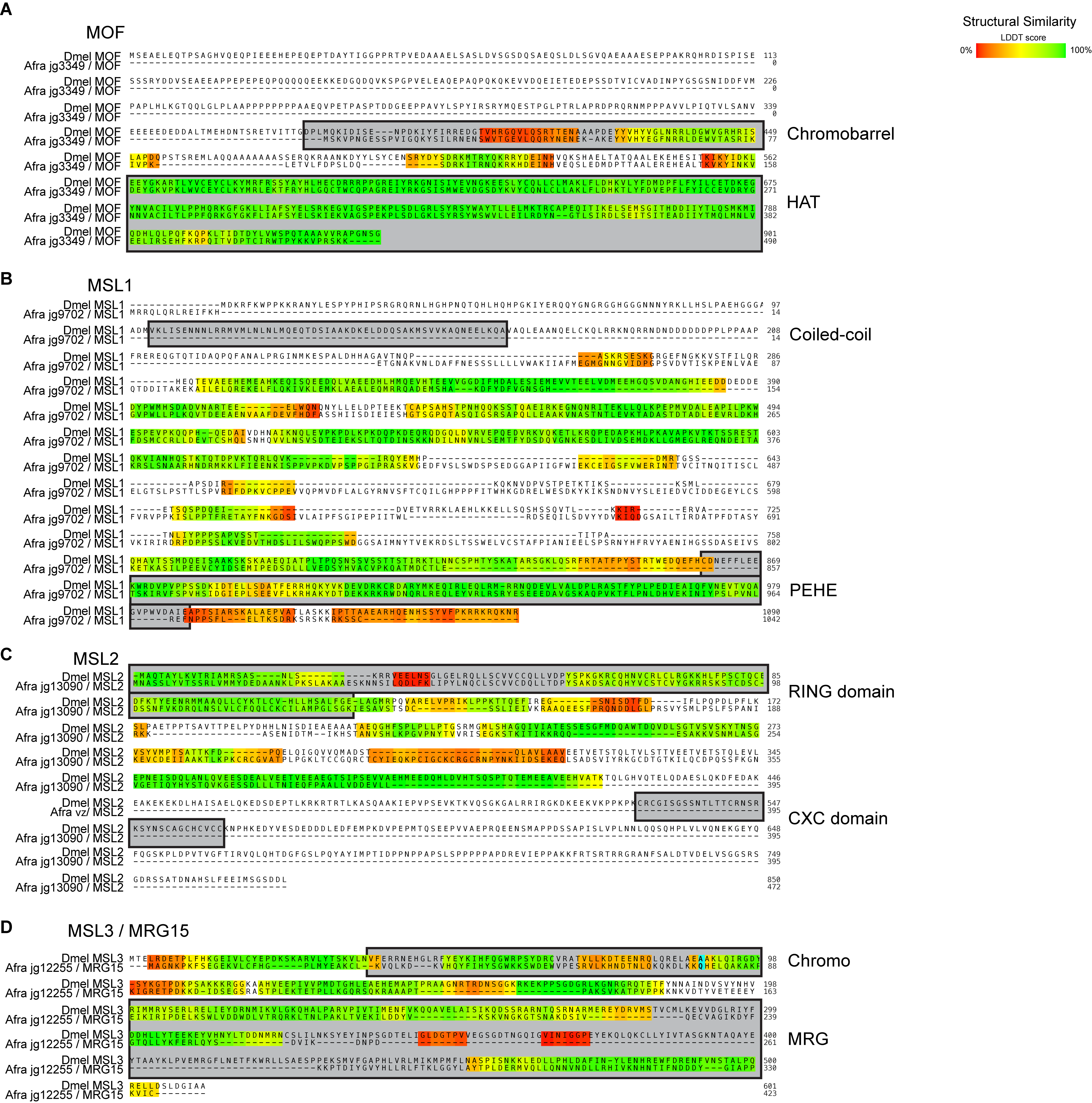

Supplement: S4 Fig — (A) Structural similarity alignment of MOF orthologous protein sequences from A. franciscana and D.melanogaster, with the chromobarrel and HAT domains highlighted in a box. Structural similarities were assessed using FoldMason and are presented as Local Distance Difference Test (LDDT) scores. (B) as in (A), but for MSL1 orthologous protein sequences, highlighting the coiled-coil and PEHE domains. (C) as in (A), but for MSL2 orthologous protein sequences, highlighting the RING and CXC domains. (D) as in (A), but for MSL3/MRG15 orthologous protein sequences, highlighting the chromo and MRG domains. (TIF) [file pgen.1011895.s004.tif]

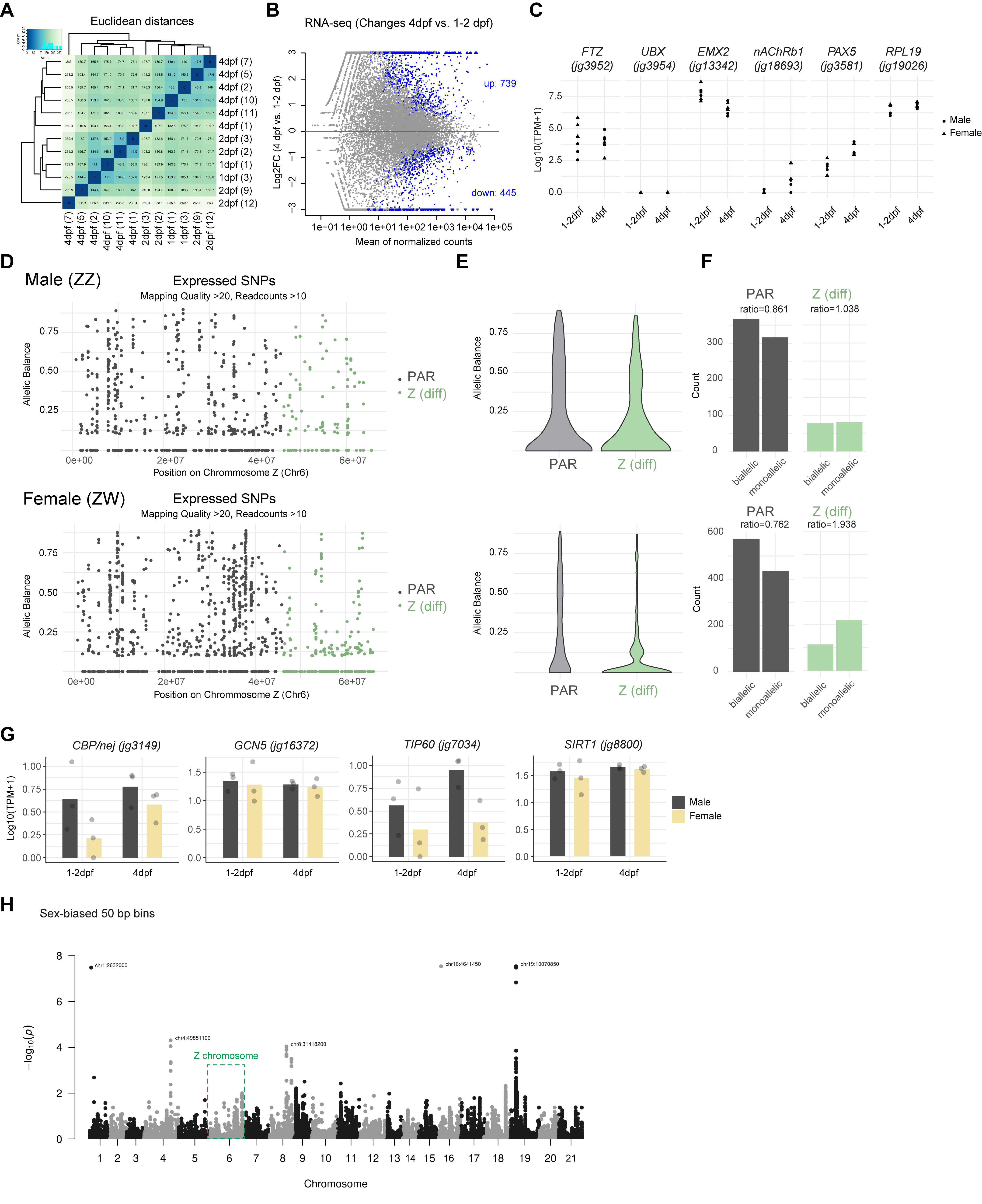

Supplement: S5 Fig — (A) Hierarchical clustering of embryonic low-input RNA-seq samples was performed using Euclidean distance calculations based on rlog-transformed counts in DESeq2. A distance matrix was computed from the rlog-transformed expression values, and a heatmap was generated using the heatmap.2 function from the gplots package in R. (B) MA plots showing differentially expressed genes obtained by DEseq2 between early embryonic stages (1–2 dpf) and later embryonic stages (4 dpf). The plot shows mean normalized counts (x-axis) against log2FC (y-axis), with significantly changing genes FDR < 0.05 colored in blue. (C) Dot plot showing gene expression values as Log10(TPM + 1) for selected genes in the two stages. Circles (males) or triangles (females) show the values of the individual replicates. (D) Dot plots showing allelic balance (y-axis) obtained by FreeBayes of exonic SNPs along their position on the Z chromosome (x-axis). SNPs were filtered for locating in exons, mapping quality >20 and minimum coverage >10. The plots show the results from one adult male (top) and one adult female (bottom) dataset, with the SNPs residing in the Z(diff) region colored in green. (E) as in (D), Violin plots displaying the distribution of allele frequencies of each exonic SNP, comparing the pseudoautosomal region (PAR) and the differentiated region of the Z chromosome in males (top) and females (bottom). (F) as in (D), bar plots comparing the number of biallelic (allelic balance > 0) and monoallelic (allelic balance = 0) SNPs in the PAR and the differentiated region of the Z chromosome. (G) Bar plots showing the mean RNA levels (n = 3) of histone acetyltransferase (CBP/nej, GCN5, TIP60) and the histone deacetylase (SIRT1) genes along embryogenesis obtained by RNA-seq in males and females. The overlaid dots represent the individual Log10(TPM + 1) expression values of a given biological replicate. (H) Manhattan plot of differential transcript expression between male and female A, franciscana embr [file pgen.1011895.s005.tif]

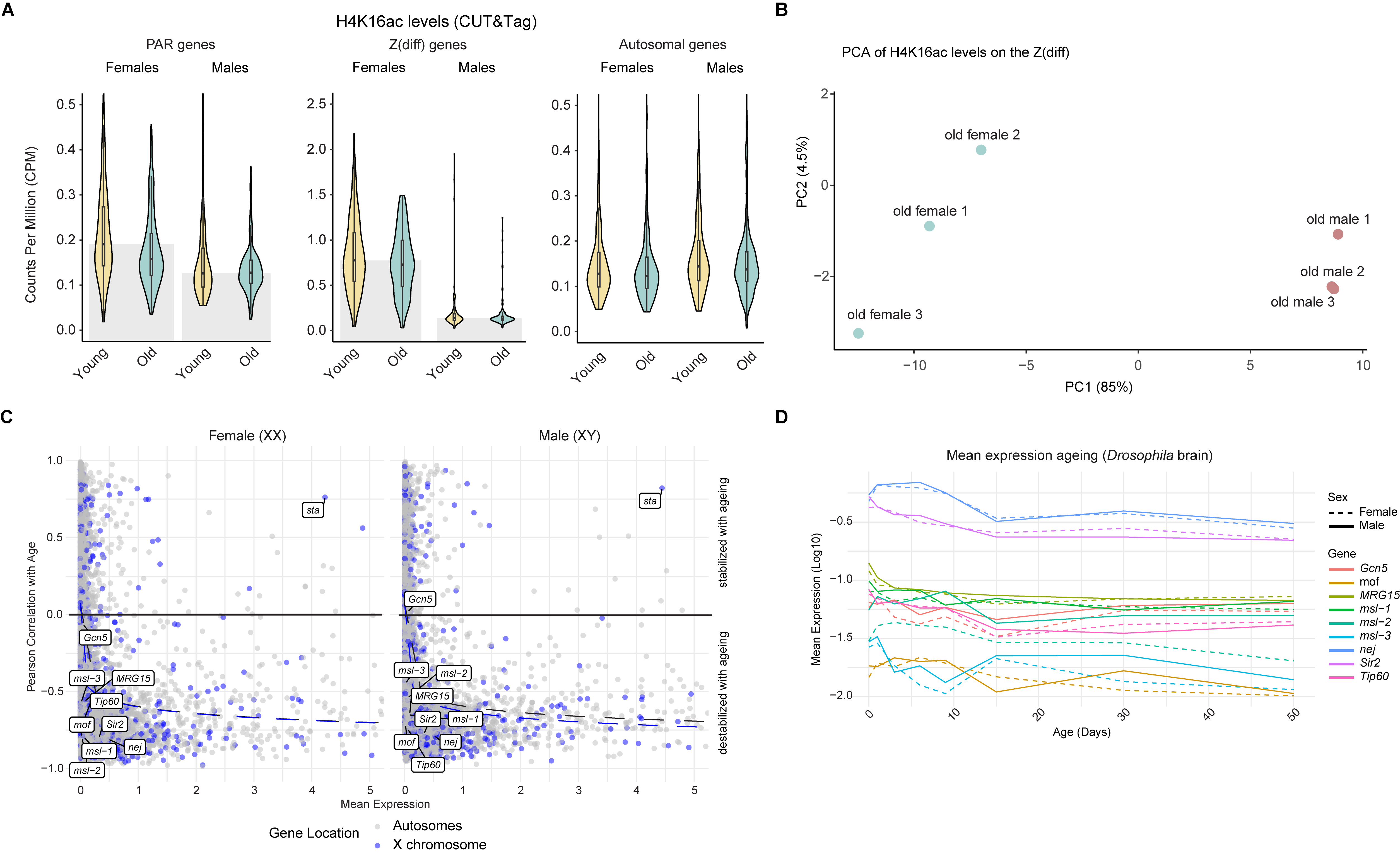

Supplement: S6 Fig — (A) Normalized H4K16ac CUT&Tag levels on expressed genes (TPM ≥ 10) in males and females, shown as a violin plot with an overlaid boxplot for each chromosomal region (autosomes, PAR, Z(diff)). H4K16ac enrichment levels for each replicate and sex from TSS-1kb until TES were calculated using deepTools multiBigWigSummary from the normalized signals in counts per million. (B) PCA of H4K16ac levels at expressed genes (TPM ≥ 10) of the differentiated Z chromosome in old (75 days) female and male samples, based on CUT&Tag profiling. Each point represents a single individual/ biological replicate. (C) Dot plot showing the Pearson correlation of mean gene expression with age (y-axis) along with the corresponding mean expression value (x-axis) for each expressed Drosophila gene in females (left plot) and males (right plot). The black horizontal line at 0 marks no decline across aging. The data is a single cell RNA-seq dataset from Drosophila brain [70] where 8 time-points until 50 days adult age are sampled. The mean expression across all cells was calculated based on the absolute expression values in each cell (UMI counts). Genes with 0 mean expression in more than half of the sampled time-points were removed. sta is an exceptional gene with increased stability upon aging (positive Pearson correlation) and is labelled as a control. MSL and histone acetylation factors are also labelled and decay upon aging (negative Pearson correlation). X chromosomal genes are shown in blue. The dashed blue and black lines represent a log linear regression for X and autosomal genes. (D) Line plot showing how log10(mean expression) of selected histone acetylation factors changes in different adult ages of Drosophila, with females shown as dashed and males as solid lines, respectively. Single-cell RNA-seq dataset from [70] as in (C). (TIF) [file pgen.1011895.s006.tif]

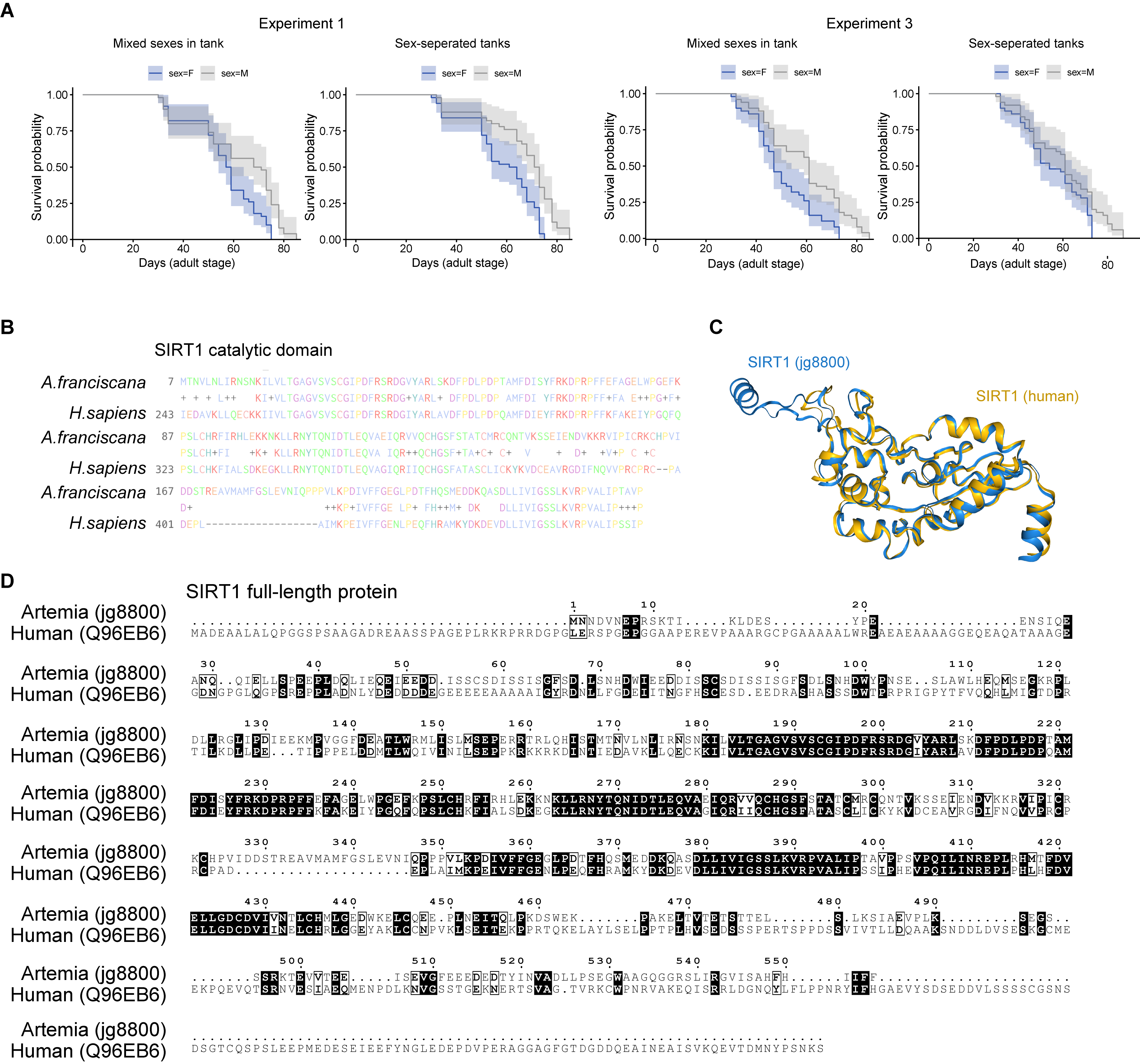

Supplement: S7 Fig — (A) Line plot showing the mean survival probability with shaded confidence intervals of male and female Artemia when they are reared (left:) in a mixed-sex culture or (right:) in separate cultures (no mating). Each replicate culture reflects 50 adults of each genotype seeded. The x-axis corresponds to the days after hatching. The experiment was started by culturing the same number of adult stage males and females (25 days) as before the sexes cannot be visually distinguished. The experiment was independently conducted three times, one more replicate is shown in Fig 6A. (B) Sequence alignment of the catalytic domain of A. franciscana SIRT1 and H. sapiens SIRT1 protein sequences (C) AlphaFold-based 3D structural similarity comparing the catalytic domain of A. franciscana SIRT1 protein (jg8800) and human SIRT1. (D) as in (B), but for the full-length SIRT1 protein sequences. (TIF) [file pgen.1011895.s007.tif]
