# Supplementary material for: Convergent evolution of H4K16ac-mediated dosage compensation in the ZW species Artemia franciscana
Source: PLoS Genet. 2025 Oct 9;21(10):e1011895. doi: 10.1371/journal.pgen.1011895 (PMC12527168; doi:10.1371/journal.pgen.1011895)
Supplement: S3 Data — (PDF) [file pgen.1011895.s010.pdf]

Genome Browser Snapshots and BLAST hits of putative lncRNAs

Tracks 1-4 show coverage in embryo RNA-seq datasets, Tracks 5-6 show adult RNA-seq datsets.

>lnc1::chr19:10071250-10071300

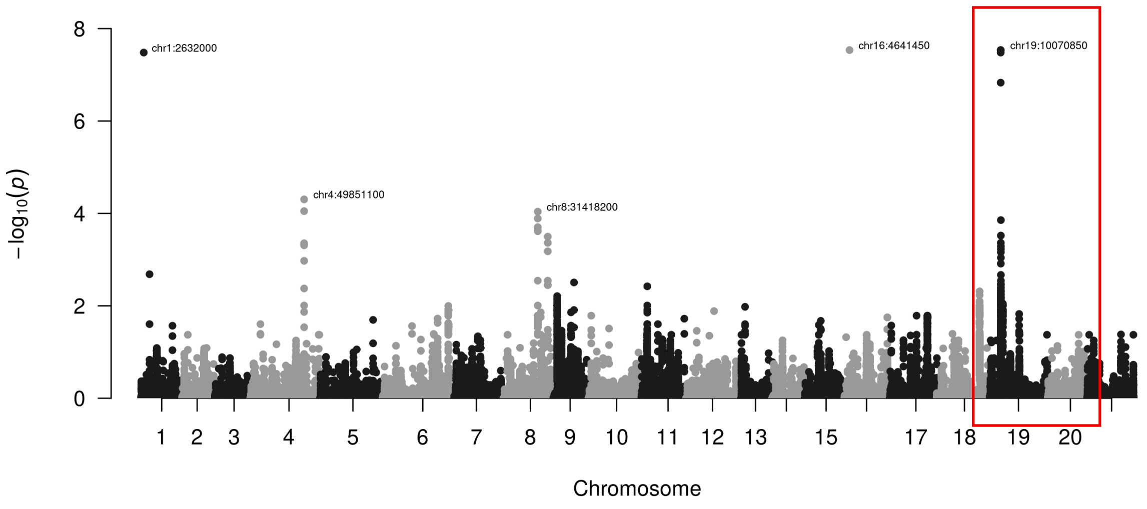

>lnc1::chr19:10071250-10071300

AATTAAAAAAAGAAGAAAATATTCTACATTGATCACGTTCACAGGAGCTT

Uncharacterized long non-coding RNA

PREDICTED: Artemia franciscana uncharacterized LOC136028966 (LOC136028966), ncRNA

NCBI Reference Sequence: XR_010617929.1

GenBank Graphics

>XR_010617929.1 PREDICTED: Artemia franciscana uncharacterized LOC136028966 (LOC136028966), ncRNA

TTGATGCCTTCAACCCTCTATACTATGTAAAATGGAAAATAACTGGTTAGTTAATAATGATCTACTGTTC

AGAATCAATTTTATATTGTACGGAGCTTATGCAAGCCAGATACGTATGCGAGATTTTCTTTGGCGAATGC

TTCTGACTAGTGCCTTGGTAACATATATATTGGATATTCTAGCCAGGATTTTAAAACTGTACTTATTACG

AAACATCACTCGAACTAATGGAAACCCGAAGACAAGCATGATGTCGCGGCTGAAGATTTAACTGACGAAG

CCGCAGAAAAGAAAGAAGCCGAGGAGACTGCGAAGCGAGACTCGAAGCCTGATTCAGGTGTAGAGGAGAC

GGGTTGTCCTGGAAGTCTTAGAAAACGAAAGTCTCGGAAACATTAAAGCTCCTGTGAACGTGATCAATGT

AGAATATTTTCTTCTTTTTTTAATTGTCTTGGTCTGATGTTTTTTGATGAAATGATCTATGTCTCTTTGG

AGCTGCCTTACTGTTTATTCAATAACTTGTGGTGTTGGCATTCTGTTTACAAAGAATTGAACAGGGCTAC

TGGAAAAGGATGCATTCTAAAATGGGAAATAACTTTTATTGAAAACAACTGAATCTTGAAAGTTTTTTTA

CGCCATATTTAAGCAGTTTTGCGACATATCTATTTATAAGACCATTAAAAATATTGGCCAAAAACTGTGT

GGGAATATGGCGCACCACTCTTTTTTTTAAAGTGAGTTTTTTCTTGCTGCTAGAAAACTAATATTATTCT

TAGTTATCTGCATTCGAATGGCCAGCTCAATTACATTTACGAAAGTGCAGTTGTATATAAGAAGGACAAT

ATAAGAGTACGTATTATTGAGAGTATTTGGTTAGGTGCTAAACTTCTGTCTTGTATCGAAGTTGTTATAC

TTTTACTATGTGTTTATATTAAAAATTGAAATGTT

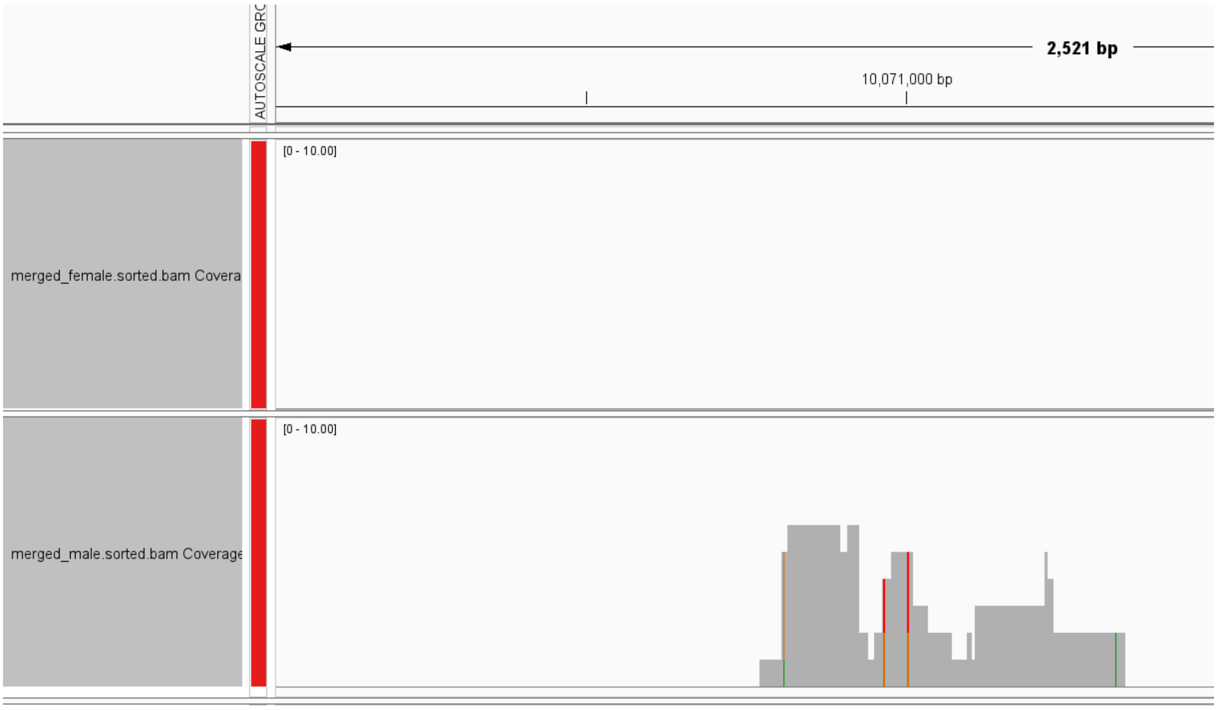

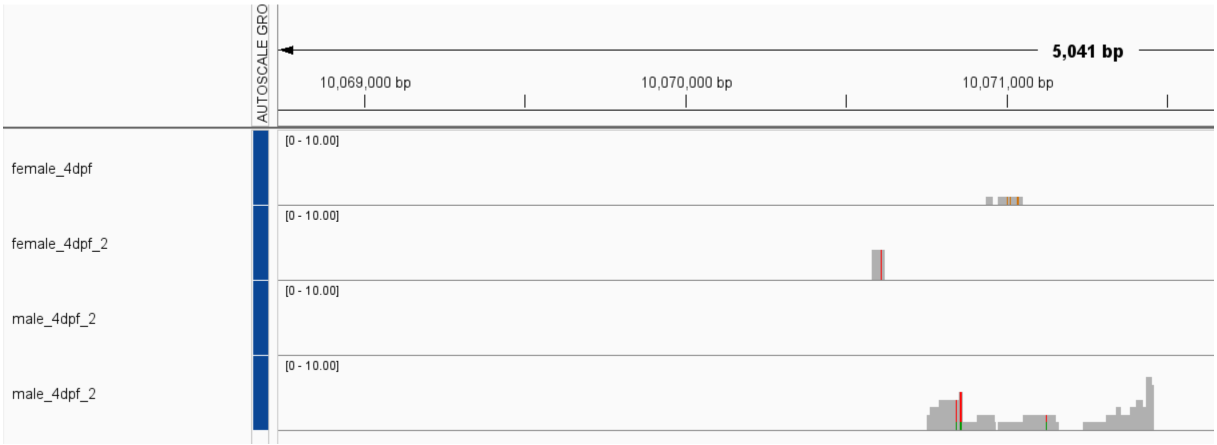

>lnc1::chr16:4641450-4641500

GAAATCCGTGAGAAACAAAATGGTGTAAAAGATTTTTGAAGACCGTGTGG

No BLAST hits obtained

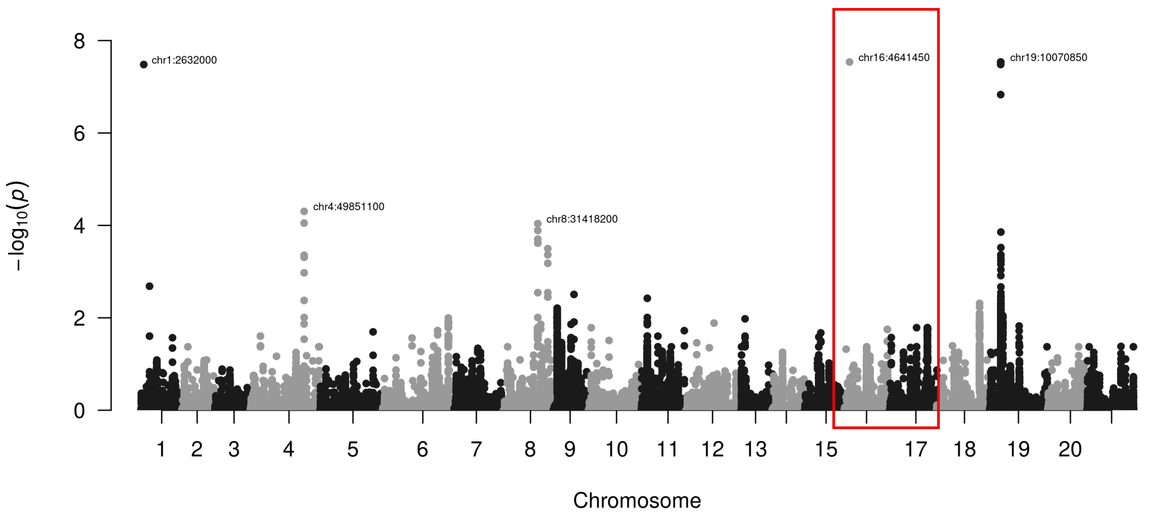

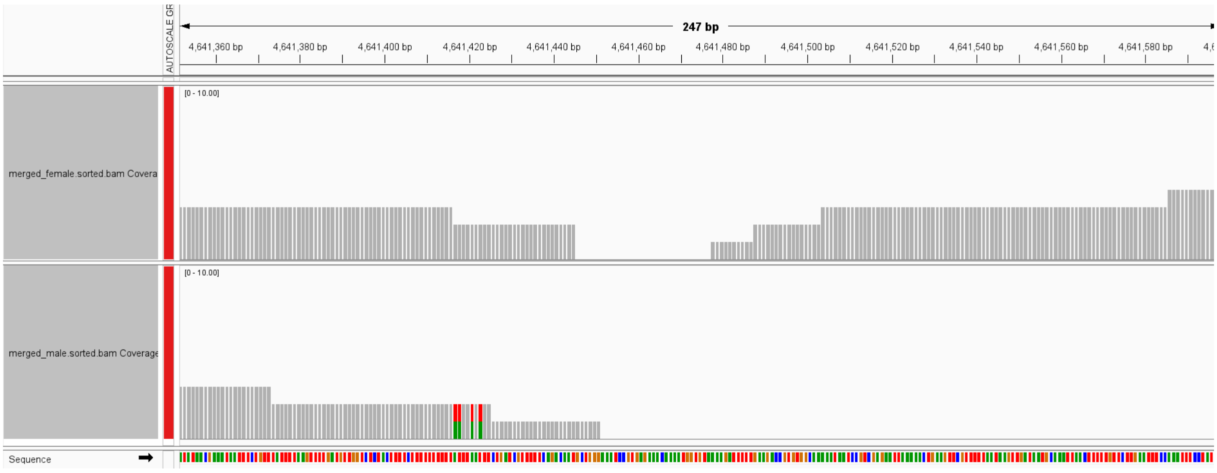

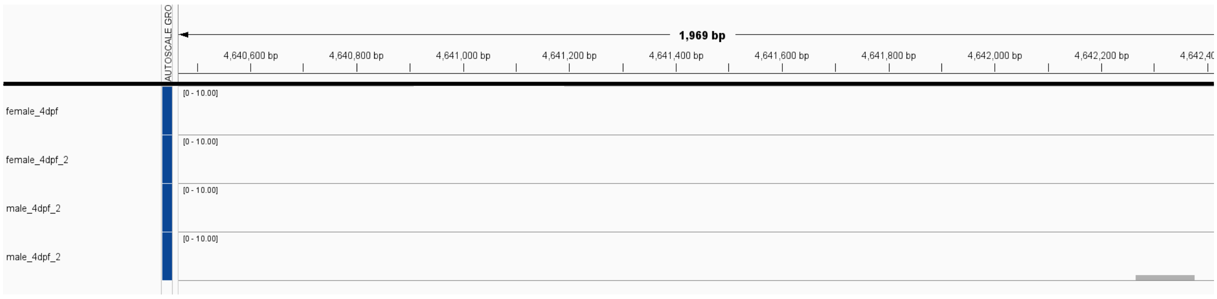

>lnc1::chr1:2632000-2632050

CTCCCAAACAGCCCTTTTAAGGGCTAACACCTTATTTCAAATGCTCTACT

PREDICTED: Artemia franciscana histone H2B-like (LOC136041918), mRNA

NCBI Reference Sequence: XM_065726716.1

GenBank Graphics

>XM_065726716.1 PREDICTED: Artemia franciscana histone H2B-like (LOC136041918), mRNA

**ATGGCTCCAAAAATTTCAGGAAAAGCAGCAAAGAAAGCTGGTAAGGCGGAGAAAAATATCAGTAAAACTGATAAGAAA
AGGAAACGAAAGAGGAAGAAAAGCTACGCCATTTACATTTACAAAGTTCTCGAGCAAGTGCATCCCGACACTGGTATTT
CTATCAAGGCAATGAGCATCATGAATAGCTTTGTCAATGATATCTTTGAAAGGATTGCTGCGGAAGCCTCTCGTCTAGCT
CACTACAACAAGAGGTCCACCATCACTAGCAGAGAGGTTCAAACTGCTGTGAGGCTGCTCCAACCCGGAGAACTTGCCA
AGCACGCTGTTAGTGAAGGCACTAAAGCTGTAGCAAAGTACACAAGCTCCAAATAAGGGGGTTTCTCCCCTCTATTGGC
GCCCAATCAGCCGGGCCCTCCCAAACAGCCCTTTTAAGGGCTAACACCTAATTTCAAATGCTCTACTCTCA**

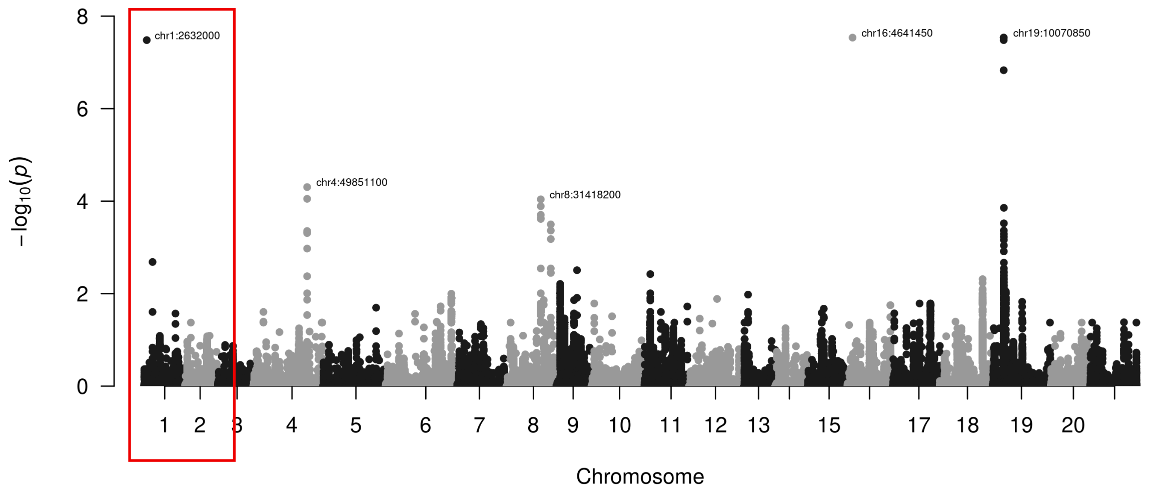

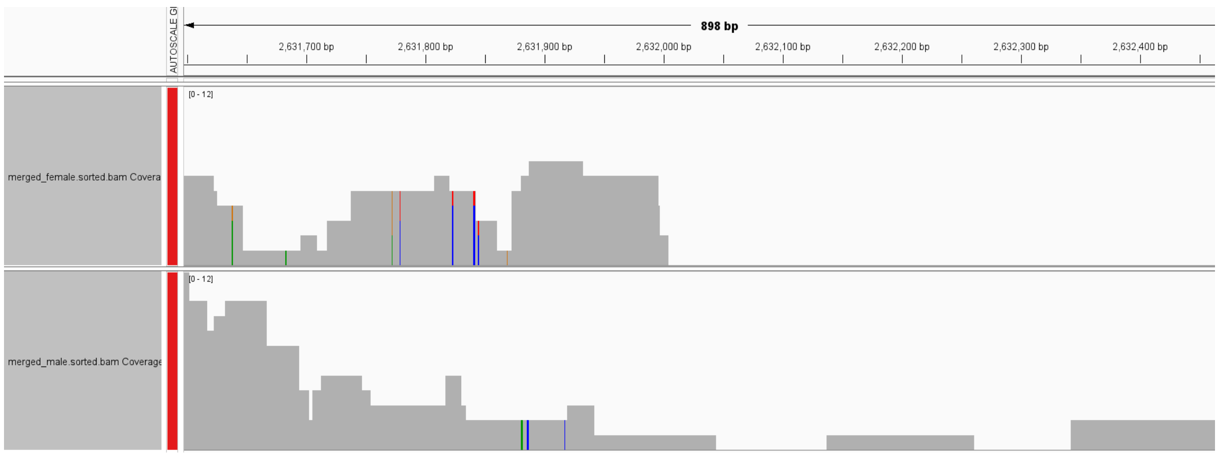

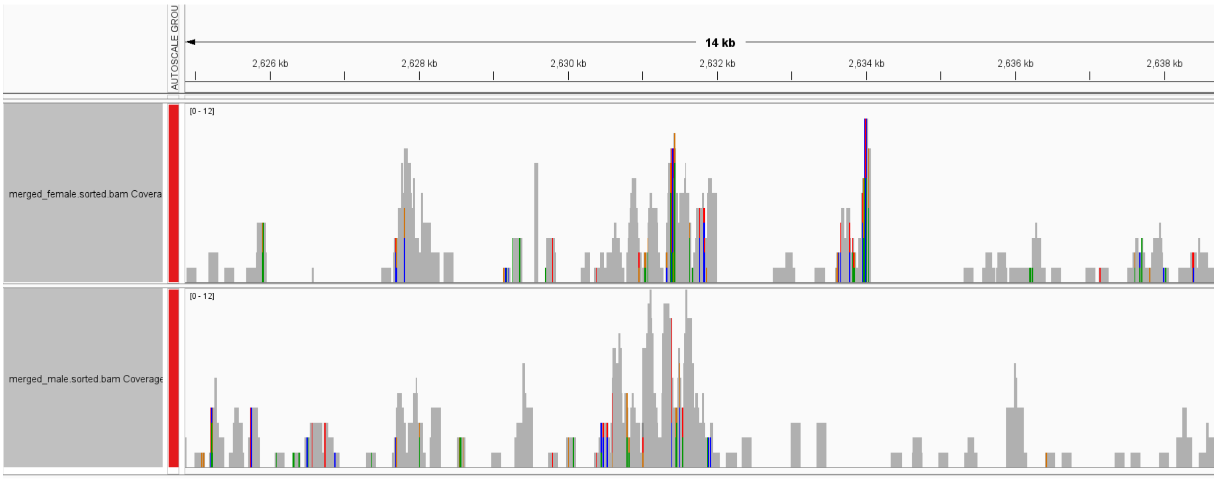

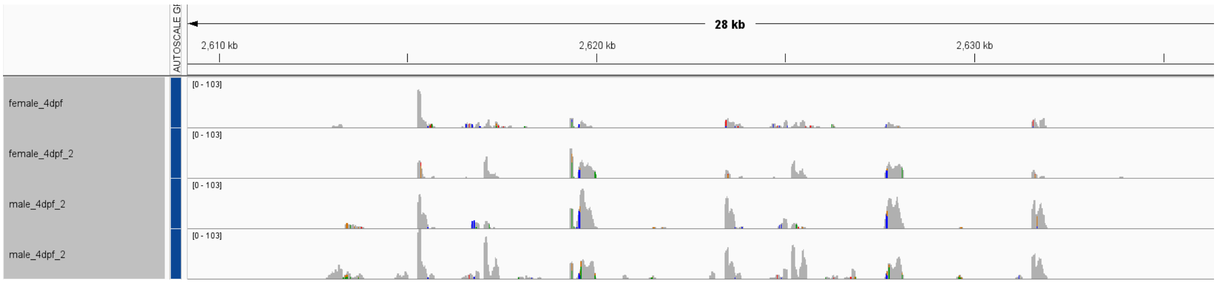

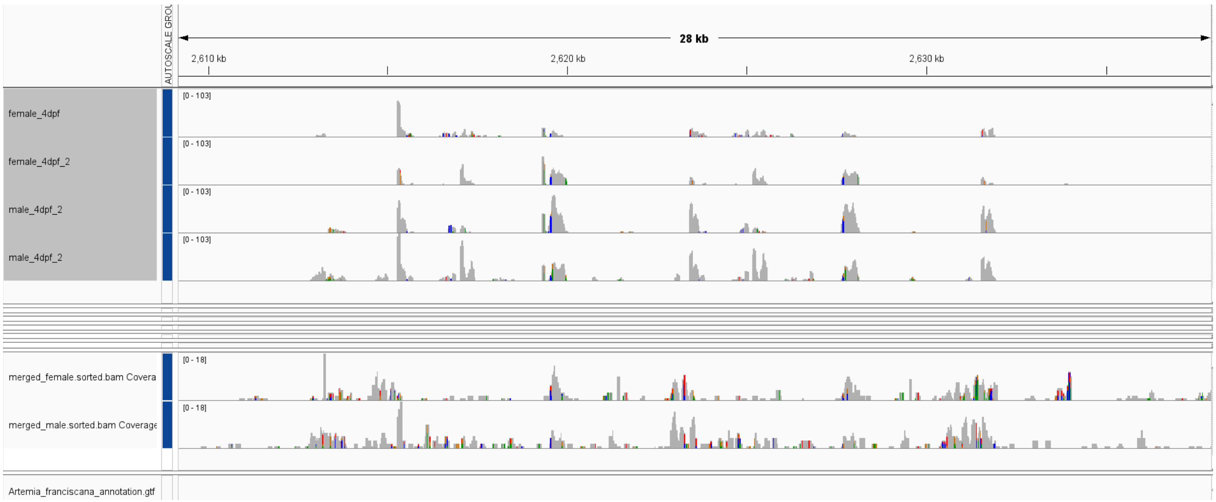

>lnc1::scaffold000243W:11500-11650

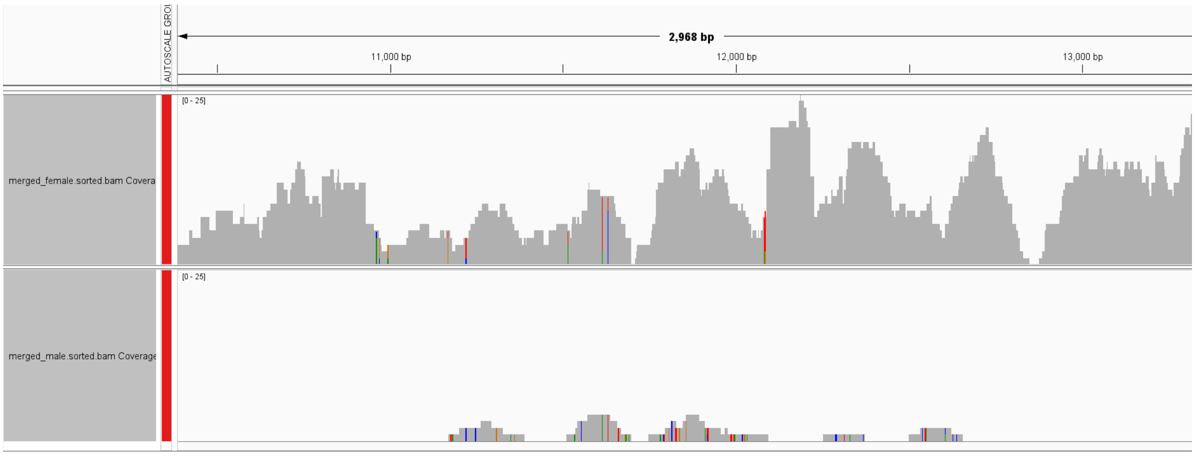

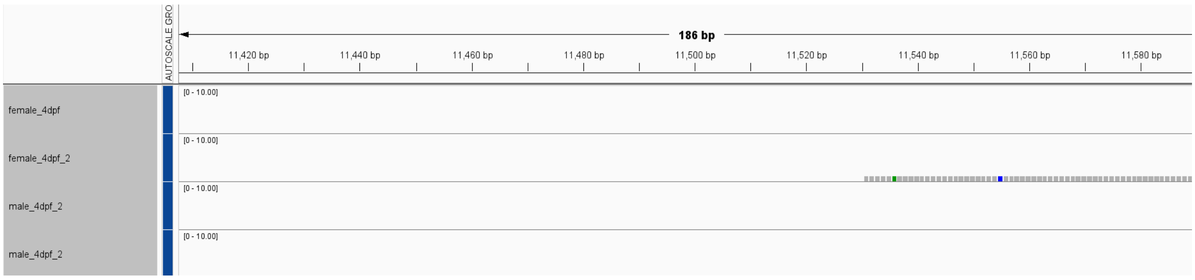

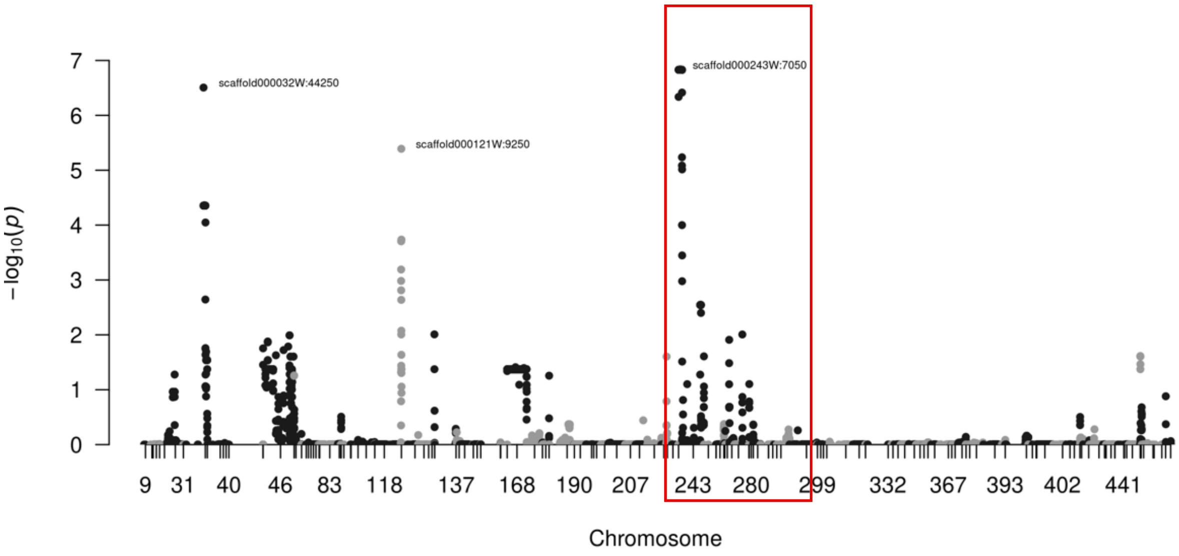

chr4:49851100

Mitochondrial 16S Ribosomal?

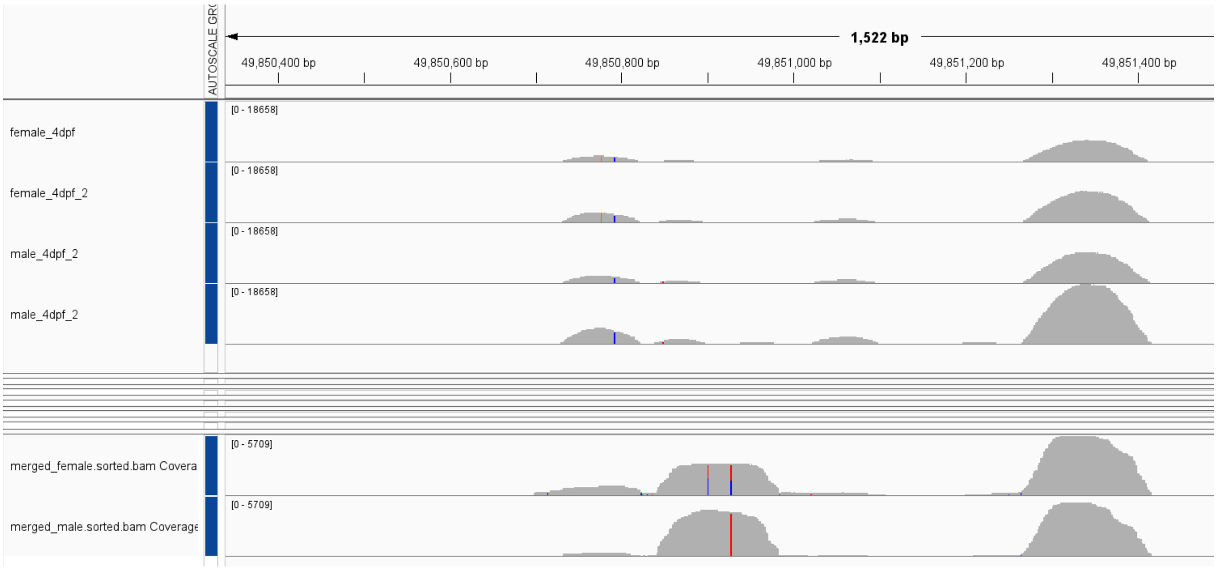

chr6:686,568-688,129

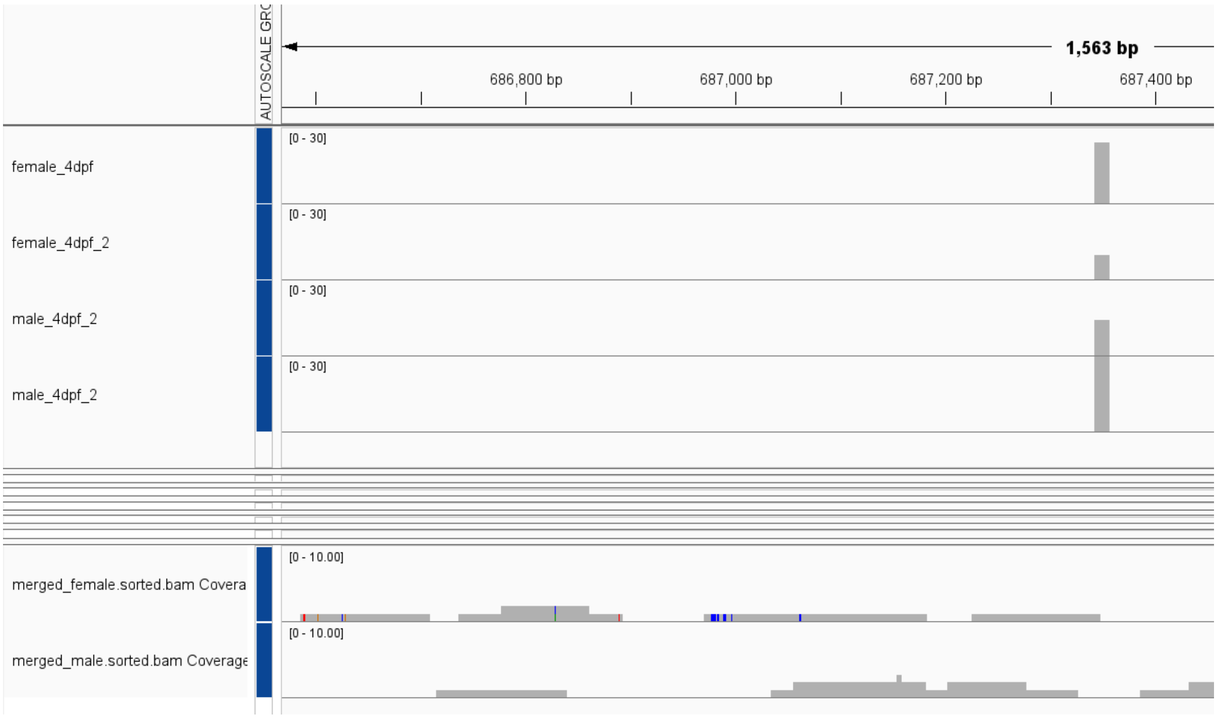

Genome Browser Snapshots of IRFinder hits (padj<0.05)

Tracks 1-4 show coverage in embryo RNA-seq datasets, Tracks 5-6 show adult RNA-seq datsets.

jg17559

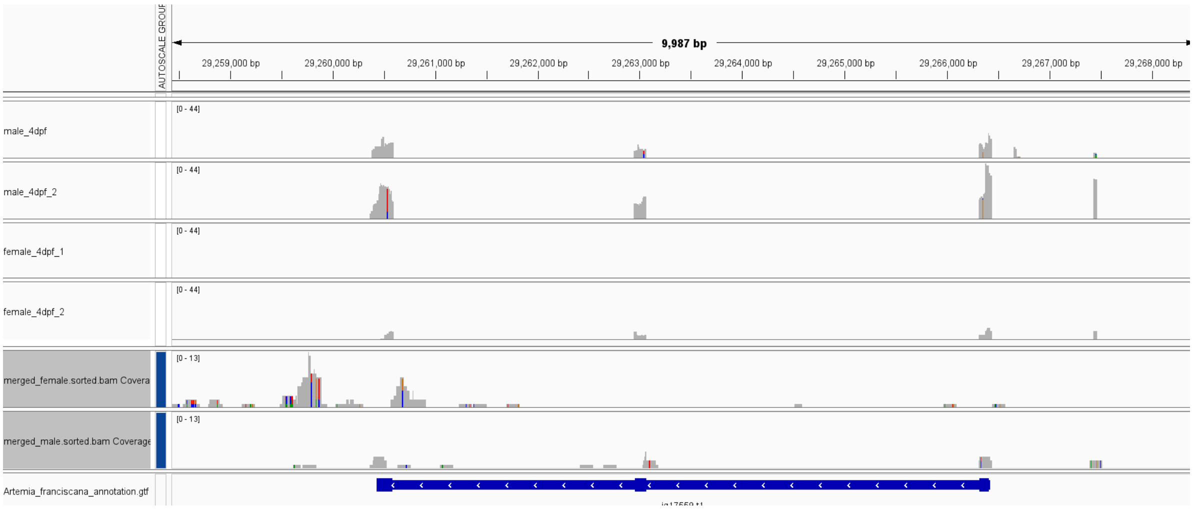

Uncharacterized protein, no domains found with prosite

jg5037

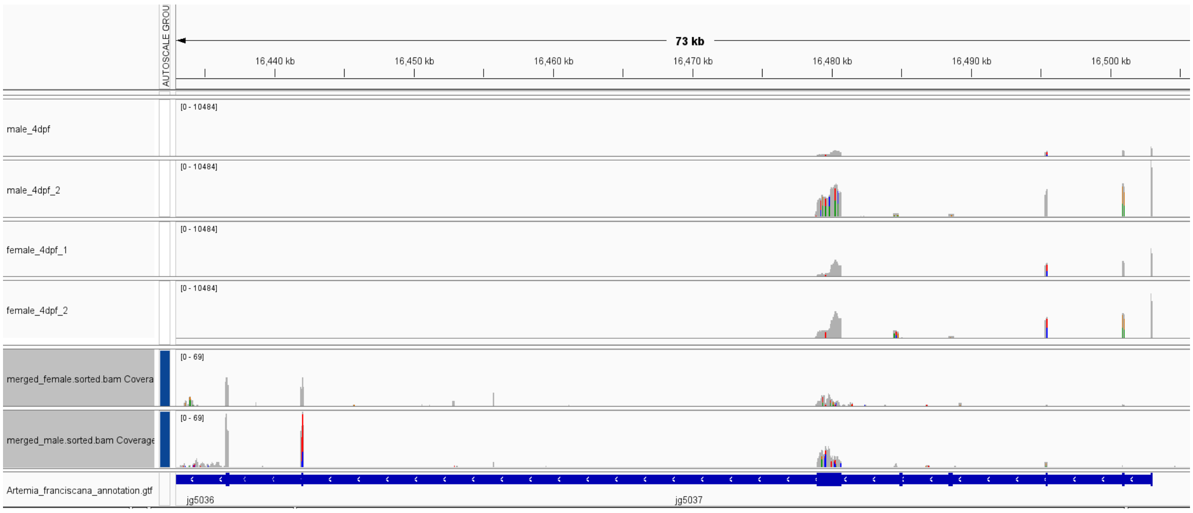

serine/arginine repetitive matrix protein 1-like

jg22222

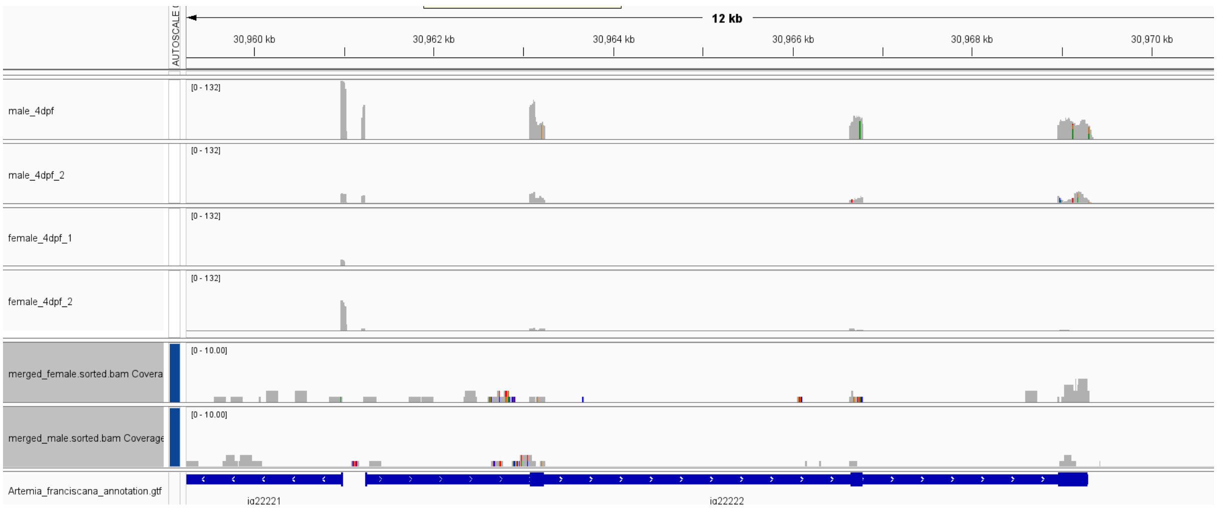

Uncharacterized protein, no domains found with prosite

jg22016

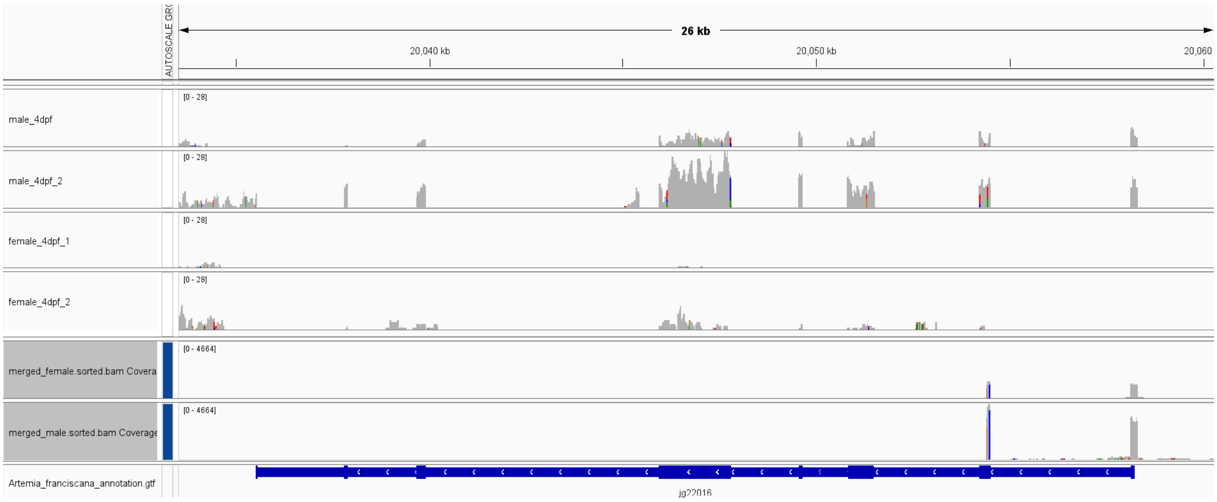

Uncharacterized protein, contains Tudor domain
