# Supplementary material for: Convergent evolution of H4K16ac-mediated dosage compensation in the ZW species Artemia franciscana
Source: PLoS Genet. 2025 Oct 9;21(10):e1011895. doi: 10.1371/journal.pgen.1011895 (PMC12527168; doi:10.1371/journal.pgen.1011895)
Supplement: Attachment — Submitted filename: 29.08.2025.Zimmer.RTR.pdf [file pgen.1011895.s013.pdf]

Response to Reviewers

Zimmer et al. - PGENETICS-D-25-00319

Reviewer #1

Overall comments

Dosage compensation/dosage balance in the evolution of chromosomal sex determination is a fascinating problem, because it seems that rapid evolution of sex determination systems goes alongside enormously diverse ways in which dosage compensation happens, from either no compensation at all through to chromosomal upregulation, chromosomal downregulation and/or inactivation. The ‘rules’ of this, if there are any, are completely unknown. The only way we can get enough information to even begin to speculate is through studying many different sex chromosomal systems to see whether each system is unique or whether there are any commonalities. In this regard, the current manuscript is a really important study because it considers dosage compensation in a ZW system (the crustacean *Artemia*), which are currently understudied at the molecular level due to lack of appropriate model systems. The manuscript thus tackles an important question. Moreover, it is largely novel - it reports chromatin-based dosage compensation through H4K16Ac, which is similar (although not identical) to the *Drosophila* dosage compensation mechanism, even though *Drosophila* is XY rather than ZW. Most of the results are presented quite well and some key questions are answered, such as the differences between pseudo-autosomal regions that are still recombining and more divergent regions; other questions, such as exactly how the H4K16Ac is recruited will hopefully open up new avenues of research for the future.

We would like to thank the reviewer for the careful analysis of our work and for their supportive comments and suggestions.

Despite these major strengths, the paper has a few weaknesses. The first is that it feels as though two rather distinct papers, one focussed on dosage compensation and one focussed on ageing, have been cobbled together. Whilst connected conceptually, the problem with this is that neither of the two areas has been explored in sufficient detail. It seems that it would have been better if one area had been more fully studied, with two separate papers eventually produced. In my opinion, the dosage compensation aspect is more thorough at present, but even this is missing some key aspects of data analysis, and would benefit from some further experiments to better characterise the system. The effect of H4K16Ac on ageing is weaker and needs more work to be convincing. I think that if the authors want to keep both of these in the same paper, they need to do more on both aspects. In the specific experimental/analytic comments below I have some suggestions for what I think needs doing to make the paper stronger.

Major points

1) The authors identify the *Artemia* system as one where K16Ac is important on the basis of chromosome staining followed by ChIP. This is convincing. However, this approach cannot rule out that other species which do not show a K16Ac territory do not have K16Ac sex-chromosome dosage compensation - it is not necessarily the case that a territory is always present whenever K16Ac enrichment on the sex chromosome is present. It would be good if the authors, as a control, could do ChIP on a system that they would predict would NOT have sex chromosome K16Ac, and confirm their prediction. This would justify the screening approach as a way to identify potential K16Ac systems.

We have now performed genome-wide profiling of H4K16ac in the stick insect *Bacillus grandii*, a species that did not exhibit a sex-chromosome territory in our microscopy, along with *Drosophila melanogaster* (positive control with a territory). Additionally, we provide plots of our previously published H4K16ac ChIP profiles (Keller Valsecchi et al., 2021) for *Anopheles gambiae*, which also lacks a territory. These new data and analyses (**Fig. 2A-C, Fig. S2A-B**) are in full support of our conclusion that H4K16ac does not mediate dosage compensation in *B. grandii* nor in *A. gambiae* (see also (Kalita et al., 2023; Keller Valsecchi et al., 2021)) and validate the microscopy approach.

2) The K16Ac enrichment does appear clear from the ChIP. However, there is an important concern - it is not clear from the Figure legend how the normalization in Figure 2's genome browser snapshots was carried out. Moreover, the scale shown is different between the two genotypes. Crucially, it is technically possible that the Cut and Run profile could be an artefact of higher accessibility. The authors presumably have normalized to IgG controls in the snapshot, but it would be preferable to actually show the IgG traces too, to confirm that they are not different between the two genotypes. The normalization relies on linearity, which may not be a valid assumption in this case, so if the IgG is much higher in one genotype than another, it could indicate that the K16Ac enrichment is artefactual.

We thank the reviewer for raising this point and apologize for the misunderstandings:

1. The data has been normalized to read counts in each sample (counts per million, CPM), but not to IgG. This information had been previously provided in methods, and was now added to the legend.
2. The choice of scale in the screenshot was on purpose: The enrichment in the females is so high, that visually it would be quite difficult to score anything in males, if scales were the same. The point here was to illustrate the difference in pattern, rather than extent of enrichment. For the latter (intensity differences), we would like to refer to the metaplots and boxplots (previously in Fig. 2, now **Fig. 3C**), which had been scaled the same for males and females allowing appropriate comparison.
3. While we always conduct IgG controls (the metaplots for those had been provided already in our initial manuscript, now **Fig. S2D, bottom panel**), we prefer to not normalize our data to IgG (e.g. by ratio or subtractions) but showing it as a separate track (as also preferred by the reviewer).

4. As suggested, we have revised the genome browser snapshot including now also the IgG tracks (now **Fig. 3D**). Please note that the background is really low/sparse, which is typical for CUT&Tag methodology.

Together with the new histone modification profiles, the microscopy provided in Fig. 1 and the independent validation of our findings by the Vicoso group (Bett et al., 2025), this clearly demonstrates that H4K16ac enrichment on Z-diff is not artefactual.

3) A very important point is that the primacy of K16Ac in the dosage compensation cannot be established without understanding the mechanism of recruitment. The authors do not find any evidence of sex-specific K16Ac complex expression, and cannot show increased recruitment of the Acetylation complex. Histone modifications obviously correlate, so some other mark may be the driver of the K16Ac. An alternative, perhaps less likely but very interesting possibility is that there is a two-tier system happening here: it could be that there is downregulation of the entire chromosome by increased repressive histone marks, but then in the heterogametic sex upregulation by K16Ac occurs on top. In an ideal world it would be nice if the authors could do four further ChIPs which would give a bit more insight by covering major marks implicated before in dosage compensation in other systems:

H3K4me3

H3K9me2

H3K27me3

H3K36me3

Whilst this is a fair amount of work, I think it would be important if the authors wish to argue that this is an example of a K16Ac dominated dosage compensation system, rather than something more complex. For example, if there were similar expansion of H3K4me3 beyond promoters, this might suggest that it too is important in the dosage compensation (this doesn't happen in *Drosophila*); if there is also enrichment of silencing marks either in the homogametic sex or in both sexes that might suggest a more complex, two-tier system is in operation.

The possibilities that the reviewer raises are exciting. To our help came that the Vicoso lab has - during the course of our revision - just published a chromatin analysis in *Artemia* (Bett et al., 2025) including also the marks requested by the reviewer. We have used their data to investigate the questions of the reviewer.

On a global level, H4K16ac is by the far the most dominant, female-enriched mark on Z(diff) and thus - considering the microscopy and what is known about DC and H4K16ac in other species - likely the primary cause to DC (new **Fig. 3E**).

The quantification / statistical analysis shows (new **Fig. 3F**):

- Difference on the Z(diff) between males and females is biggest for H4K16ac.
- H3K27me3 appears slightly enriched on PAR and Z(diff) but this effect is visible in males *and* females.

- H3K4me3, H3K36me3 (activating) and H3K9me2 (repressive) appear slightly depleted from Z(diff) in females, while it remains the same in males. This is interesting, because this could contribute to fine-tuning of the approximately 2-fold effect necessary for compensation.

This new analysis along with discussion has been added to the manuscript.

4) The ageing aspect of the paper is very interesting, but at present over-interpreted. The key problem is that the experiment with nicotinamide is not strong. There is no evidence directly presented that nicotinamide changes H4K16Ac levels. Without this it's impossible to be certain that this inhibitor's effect on ageing is anything to do with SIRT1 or sex chromosome dosage at all. If they want to argue this then they need to do ChIP after nicotinamide treatment, and also show that changes in gene expression correlate strongly with areas where the nicotinamide treatment shows the largest effects.

The critique regarding the nicotinamide experiment is very valid (also see reply to the same point raised by Reviewer#2 and scheme in **Reviewer Fig. 1A**).

For revision, we have performed CUT&Tag (2 replicates each, along with IgG controls) with the following antibodies: (A) H4K16ac and (B) pan-acetylated H3 with the following samples: (1) adult males and females (treated with Control / Nicotinamide for 24 hours) and (2) late embryos (eggs) that were freshly hatched to Naupli in either Control / Nicotinamide containing water (duration of treatment ~24h). The results were interesting but also in parts unexpected (see following **Reviewer Fig. 1**).

In support of our earlier data presented in the manuscript, we find:

- Adults: H4K16ac levels change at promoters / TSS upon Nicotinamide treatment, with a much more pronounced effect compared to H3ac, supporting specificity (**Reviewer Fig. 1B**, left and middle panel).
- Adults: Responses for both H4K16ac and H3ac are stronger in males than in females, suggesting that females could be more resistant to HDAC modulation (which we also observed in lifespan measurements, as well as RNA-seq).
- Adults: Z(diff) (green line) shows the strongest response to Nicotinamide treatment compared to autosomes (black line) or PAR (grey line).
- Naupli: Nicotinamide changes acetylation levels at gene bodies (**Reviewer Fig. 1B**, right panel).

However, there are also puzzling findings:

- In adults, the observed change is a *decrease* in acetylation at promoters (**Reviewer Fig. 1B**). Given that Nicotinamide is expected to inhibit HDACs, we would expect an *increase* (**Reviewer Fig. 1A**).
- At the Naupli stage, we do observe the expected *increase* in acetylation (**Reviewer Fig.1B and 1C)**, but here we cannot distinguish sexes, since the experiment was done on pooled individuals (these are too small to yield sufficient material for single-individual CUT&Tag, while sexes are morphologically indistinguishable). In addition,

we have no corresponding RNA-seq to be able to score corresponding responses at gene expression level.

- We have no explanation to why Nicotinamide primarily affects the promoter regions in adults, while it affects gene bodies in Naupli.

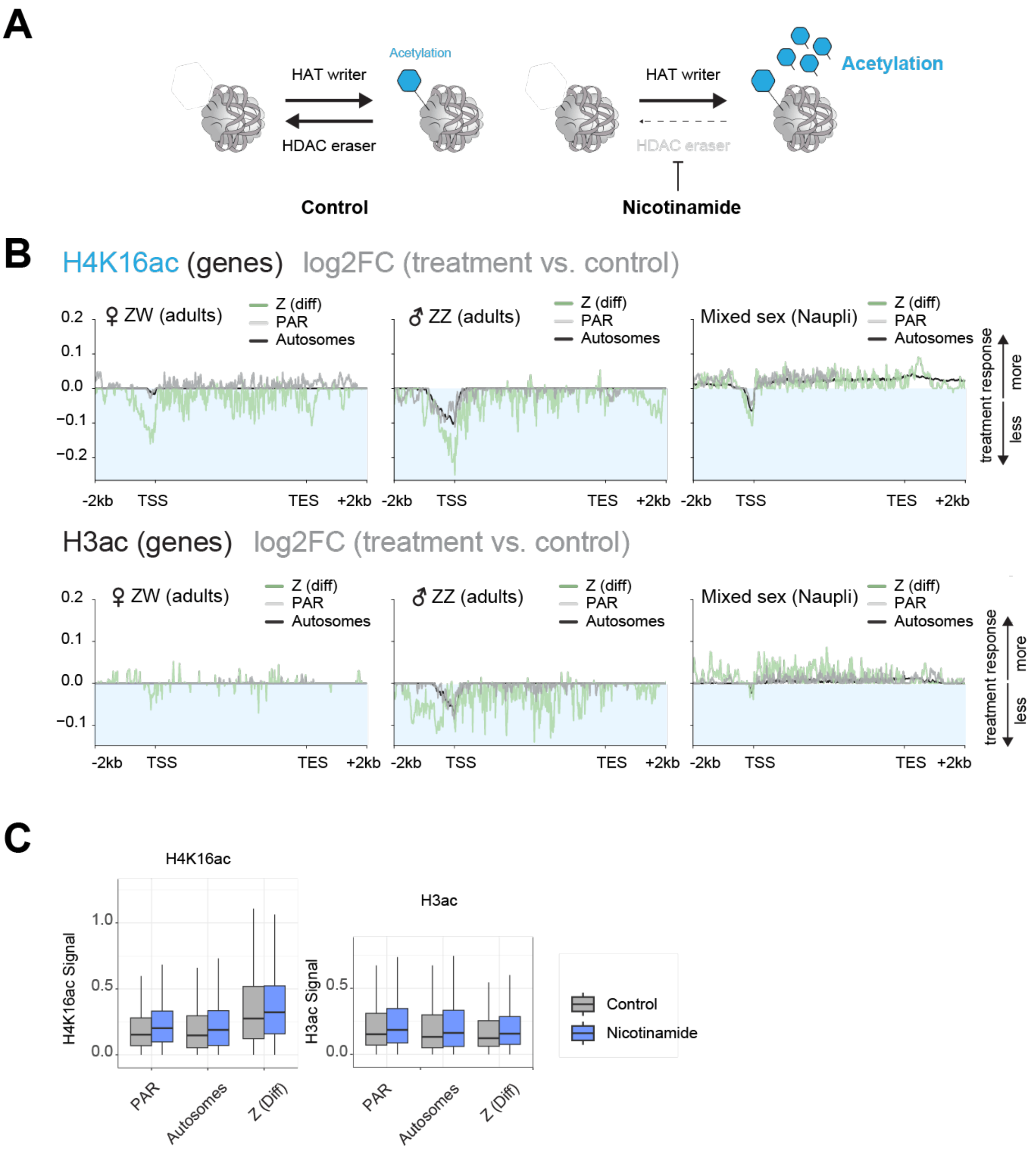

Reviewer Figure 1: Histone Acetylation changes upon Nicotinamide Treatment. (**A**) Scheme illustrating the expected impact of HDAC inhibitor treatment on histone acetylation levels (**B**) Metaplots from H4K16ac (top) or panH3ac (bottom) CUT&Tag data showing the median log2FC (treatment vs. control) for all expressed genes (TPM ≥10) on the indicated chromosomal regions. A flat line at 0 indicates equal enrichment in control and treatment, while signal below (less enrichment in treatment) or above (more enrichment in treatment) delineate changes. (**C**) Boxplots showing the read-count normalized CUT&Tag enrichment for H4K16ac and H3ac in freshly hatched Naupli (mixed sexes). The chromosomal enrichment for the different regions were calculated for equally sized bins irrespective of annotation (genes/intergenic regions are included).

These results are consistent with our (and the reviewer's) earlier speculation of a compound regulatory system in which upregulation (acetylation) and dampening (deacetylation) could occur in different Z regions concomitantly. However, these new data also raise more additional questions than they currently answer, and will require further targeted experiments.

For this reason - and in line with editor and reviewer recommendations - we have removed the Nicotinamide-treatment data from the manuscript. We nevertheless believe that the analysis of dosage compensation across life stages (embryos, adults, and ageing adults), together with the many new additions, provides a strong standalone contribution - even without perturbation data.

Minor points

The manuscript is generally well written but some of the figure callouts need checking- I noticed that Fig 2 is referred to as Fig 1 in some parts of the manuscript.

Please apologize, we have corrected the mistakes with regards to Figure labelling and callouts in the revised manuscript.

Also, the use of the word 'vulnerability' in the abstract is not very clear- it would be better to rephrase the sentence as:

'They highlight the potential role of sex-specific histone acetylation in driving male-female differences in ageing' or something similar.

We have amended the abstract, taking into account also the comments of the other reviewers.

Reviewer #2

The work by Frederic Zimmer and colleagues reports the identification of the dosage compensation (DC) mechanism acting on the sex chromosomes of the crustacean *Artemia franciscana*, a female-heterogametic (ZW) species. Sex chromosomes often show marked structural heteromorphism, leading to a fundamental imbalance in the dosage of sex-linked genes—one sex carries two copies of the chromosome, while the other has only one. To resolve this imbalance and ensure equal gene expression between the sexes, many organisms have evolved chromosome-wide, epigenetic-based dosage compensation mechanisms. However, the molecular machinery underpinning DC is well-characterized in only a few model systems.

In the first part of the paper, Zimmer and colleagues report the remarkable discovery that *Artemia* has independently evolved an H4K16ac-based mechanism of dosage compensation, convergent with that seen in *Drosophila*. This section is of very high quality—the data are compelling, the analyses are thorough, and the conclusions are well supported. The findings represent a significant and high-impact contribution to the field of sex chromosome biology.

We would like to thank the reviewer for praising the high quality of our data and judging the paper a significant and high-impact contribution to the field of sex chromosome biology.

The second part of the manuscript explores the intriguing hypothesis that dosage compensation mechanisms may create sex-specific epigenetic vulnerabilities later in life, potentially contributing to sex differences in longevity. This idea is novel and certainly worth exploring.

Thanks for the compliments. Indeed, we also think that this is an interesting and novel idea.

However, the current evidence is limited, lacks functional validation, and suffers from several important caveats. In fact, one could reasonably draw the opposite conclusion from the data: that dosage compensation is robust and remains stable with age, unlike other parts of the genome.

For instance, the choice of 75 days as the “old” time point may be too late to detect causal aging processes, as animals at this stage are likely to be terminal. H4K16ac levels on the Z chromosome do not exhibit significant age-related changes (Fig. 4C), and age-associated gene expression changes are not restricted to the Z chromosome (Fig. 4E). Interestingly, the Z(diff) genes examined show a slight increase in expression, which contradicts expectations under a model of dosage compensation erosion - reduced H4K16ac would typically be associated with decreased expression. Furthermore, nicotinamide treatment alters gene expression more broadly on the PAR and autosomes than on the Z(diff) region in females (Fig. 5D), suggesting that the Z chromosome is not uniquely vulnerable. Taken together, these findings do not support the conclusion that Z-linked dosage compensation creates a specific epigenetic vulnerability during aging.

In summary, this study presents highly valuable and novel data that merit publication. However, several of the claims of the second part of the paper—particularly those made in the title, abstract, and later parts of the manuscript—should be revised to reflect the preliminary nature of the evidence.

Overall, I strongly support the publication of this work in PLOS Genetics, pending these revisions.

We thank the reviewer for their thoughtful critique and now provide a substantially revised version taking into account the raised issues. We hope that the reviewer agrees with us that the manuscript is now ready for publication.

Please find below my detailed comments and suggestions:

Major comments:

Line 242 – Paragraph: Alterations of H4K16ac on the Z chromosome of old female

The rationale for selecting 75 days as the "old" time point is unclear and appears unjustified. At this stage, over 95% of the population has already died, with a median lifespan of 45 days. This time point is therefore far too late to capture biological events that drive the ageing process. Instead, it is likely to reflect late-stage, indirect, or downstream consequences of ageing. To properly assess the dynamics of H4K16ac acetylation on the Z chromosome during ageing, a time-course analysis using immunofluorescence staining should be conducted at intermediary ages. Suggested time points could include 30, 40, 50, and 60 days. For the findings to be relevant to the ageing process, any observed changes should occur before the median lifespan.

We thank the reviewer for this suggestion. We have now performed additional microscopy analyses and quantifications at the 55-day timepoint (referred to as “Intermediate”; new **Fig. 5B**). While we acknowledge the value of a more detailed time-course (e.g., 40, 50, 60 days), our results show that the phenotype at 55 days already closely resembles that at 75 days (“Old”), while being clearly distinct from the “Young” state. Given the clear and statistically significant differences already observable at 55 days, we hope that this adequately addresses the reviewer’s concern, especially considering the constraints of the short revision timeframe where a more fine-grained analysis (setting crosses, sample collection at all time points, microscopy and quantification) was not feasible.

Line 251 – “there was a declustering indicated by a significant increase in the mean and maximum distance between H4K16ac foci in old females.”, and Line 253-5 – “This revealed that the average levels of H4K16ac on genes on the Z(diff) region and autosomes remain similar when individuals age.”

I find it difficult to reconcile the CUT&Tag data, which show no temporal changes in H4K16ac levels, with the immunofluorescence results. Could the authors elaborate on the biological significance of the observed “declustering”? The CUT&Tag results suggest that there is no de novo acetylation of genomic regions outside the Z chromosome. What, then, do these newly

observed acetylation clusters correspond to? Could they reflect a deterioration in the spatial organization of the Z chromosome territory within the nucleus, occurring independently of changes in acetylation levels? If so, is this effect specific to the Z chromosome, or does it point to a broader disruption of nuclear architecture? To address this, could the authors assess nuclear organization more broadly using markers for other nuclear compartments (e.g., the nuclear lamina or nucleolus), or ideally, perform FISH targeting both the Z chromosome and representative autosomes?

We thank the reviewer for this interesting question. Indeed, we think that there is a general deterioration of chromatin and nuclear organization upon ageing. To substantiate this hypothesis, we have performed the suggested Lamin staining, investigated nuclear volume and heterochromatin changes (as assessed by H3K9me3 staining). We find a loss of heterochromatin (H3K9me3) and Lamin, along with an increase in overall nuclear volume. These finds are in support of a broader disruption of nuclear architecture, likely explaining why the H4K16ac foci change (new **Fig. 5C-D**)

A more extensive analysis was not feasible within the short timeframe of this revision - for example, using FISH, which would have required developing an entirely new technique and protocol from scratch. This is far from a trivial undertaking and would have been further complicated by the fact that many regions of the Z chromosome still exhibit substantial homology, making them unlikely to be distinguishable using FISH probes or chromosome-painting approaches.

Line 317 – “more autosomal and PAR genes were affected in females.”, and lines 329-331 – “Taken together, interfering with the acetylation balance affects gene expression on sex chromosomes differently in the two sexes and this is associated with male-female lifespan differences.”

These are overstatements that need to be corrected. First, no direct link between H4K16 acetylation and nicotinamide treatment is presented. The authors should assess H4K16ac levels in males and females following nicotinamide exposure.

This critique regarding the connection of H4K16ac and nicotinamide experiment is very valid (also see reply to the same point raised by Reviewer#1).

For revision, we have performed CUT&Tag (2 replicates each, along with IgG controls) with the following antibodies: (A) H4K16ac and (B) pan-acetylated H3 with the following samples: (1) adult males and females (treated with Control / Nicotinamide for 24 hours) and (2) late embryos (eggs) that were freshly hatched to Naupli in either Control / Nicotinamide containing water (duration of treatment ~24h). The results were interesting but also in parts unexpected (see following **Reviewer Fig. 1**).

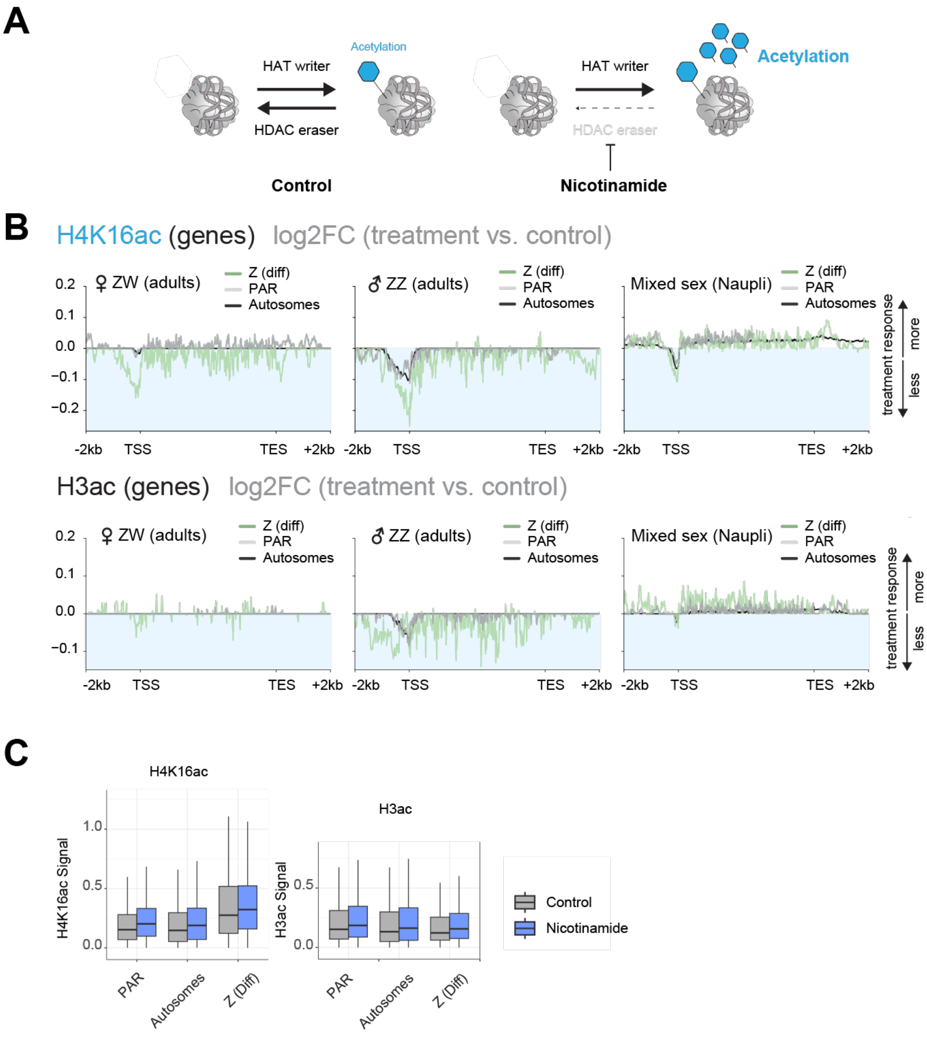

Reviewer Figure 1: Histone Acetylation changes upon Nicotinamide Treatment. (**A**) Scheme illustrating the expected impact of HDAC inhibitor treatment on histone acetylation levels (**B**) Metaplots from H4K16ac (top) or panH3ac (bottom) CUT&Tag data showing the median log2FC (treatment vs. control) for all expressed genes (TPM ≥10) on the indicated chromosomal regions. A flat line at 0 indicates equal enrichment in control and treatment, while signal below (less enrichment in treatment) or above (more enrichment in treatment) delineate changes. (**C**) Boxplots showing the read-count normalized CUT&Tag enrichment for H4K16ac and H3ac in freshly hatched Naupli (mixed sexes). The chromosomal enrichment for the different regions were calculated for equally sized bins irrespective of annotation (genes/intergenic regions are included).

In support of our earlier data presented in the manuscript, we find:

- Adults: H4K16ac levels change at promoters / TSS upon Nicotinamide treatment, with a much more pronounced effect compared to H3ac, supporting specificity (**Reviewer Fig. 1B**, left and middle panel).
- Adults: Responses for both H4K16ac and H3ac are stronger in males than in females, suggesting that females could be more resistant to HDAC modulation (which we also observed in lifespan measurements, as well as RNA-seq).
- Adults: Z(diff) (green line) shows the strongest response to Nicotinamide treatment compared to autosomes (black line) or PAR (grey line).

- Naupli: Nicotinamide changes acetylation levels at gene bodies (**Reviewer Fig. 1B**, right panel).

However, there are also puzzling findings:

- In adults, the observed change is a *decrease* in acetylation at promoters (**Reviewer Fig. 1B**). Given that Nicotinamide is expected to inhibit HDACs, we would expect an *increase* (**Reviewer Fig. 1A**).
- At the Naupli stage, we do observe the expected *increase* in acetylation (**Reviewer Fig.1B and 1C)**, but here we cannot distinguish sexes, since the experiment was done on pooled individuals (these are too small to yield sufficient material for single-individual CUT&Tag, while sexes are morphologically indistinguishable). In addition, we have no corresponding RNA-seq to be able to score corresponding responses at gene expression level.
- We have no explanation to why Nicotinamide primarily affects the promoter regions in adults, while it affects gene bodies in Naupli.

These results are consistent with our (and the reviewer's) earlier speculation of a compound regulatory system in which upregulation (acetylation) and dampening (deacetylation) could occur in different Z regions concomitantly. However, these new data also raise more additional questions than they currently answer, and will require further targeted experiments.

For this reason - and in line with editor and reviewer recommendations - we have removed the Nicotinamide-treatment data from the manuscript. We nevertheless believe that the analysis of dosage compensation across life stages (embryos, adults, and ageing adults), together with the many new additions, provides a strong standalone contribution - even without perturbation data.

Even if differences were observed, they would represent a correlation, not evidence of a causal relationship. Furthermore, nicotinamide treatment affects a greater number of autosomal and PAR genes, indicating that most of its effects are not mediated by specific deacetylation of the female Z chromosome and are likely unrelated. Finally, nicotinamide is clearly toxic to both sexes. Based on the current data, no conclusions can be drawn regarding the involvement of dosage compensation degradation in the regulation of sex differences in longevity.

The reviewer is of course right that (with the caveats explained in the previous reply) finding differences for H4K16ac upon nicotinamide treatment represents a correlation and not a causal relationship. Although we took the nicotinamide experiment now out of the manuscript (see previous reply), we would like to show the reviewer the results of another experiment that we performed for revision (**Reviewer Fig. 2**). In essence, we repeated the experiment assessing the effect of nicotinamide on lifespan with a greater number of replicates and individuals allowing us to perform statistical analysis and have better control of the variability inherent to these experiments. This substantiated our earlier claim, namely that males show shortened lifespan, while females are resistant (thus the compound is *certainly not* "toxic to both sexes" as proposed by the reviewer).

Please note that these inhibitor experiments are conducted in smaller water volumes / tanks and for this reason the absolute lifespan in days cannot be directly compared to the lifespan experiments present in the paper that were conducted in the 4L-tanks (see methods).

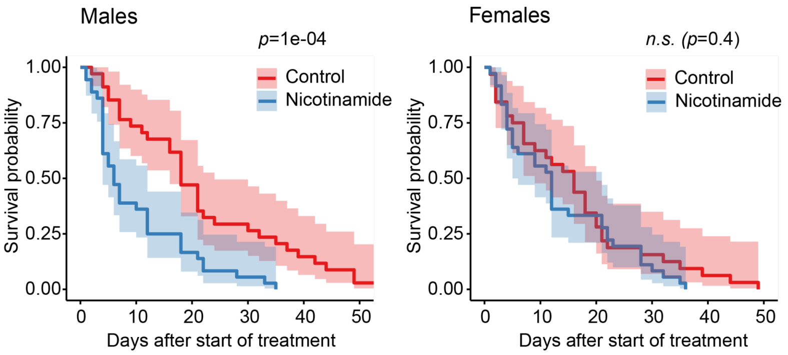

Reviewer Figure 2: Line plot showing the mean survival probability with shaded confidence intervals of male and female *Artemia* when they are reared in Control versus Nicotinamide-containing water. Each replicate culture reflects 6 adults of each genotype and sex seeded, with *n*=6 replicates performed in total for each condition (males, females, with/without treatment). The *x*-axis corresponds to the days after the start of the treatment. The *p*-value was obtained with a log-rank test for stratified data (Mantel-Haenszel test).

Minor comments:

Lines 37-9 – “along with transcriptome analysis in the "model" ZW organism, the chicken, initially led to the assumption that DC would be entirely absent in ZW systems [8] or, if present, would act only on few dosage-sensitive genes”

An important reference is missing and should be discussed here: https://doi.org/10.1101/2024.03.06.581755. In this recent study, the Kaessmann lab identified a molecular mechanism of dosage compensation in chicken. They functionally characterized a Z chromosome-linked microRNA, miR-2954, which exhibits strong male-biased expression. Knockout of miR-2954 led to early embryonic lethality in homozygous knockout males only, driven by the specific upregulation of dosage-sensitive Z-linked target genes. Moreover, they showed that miR-2954 represses the expression of approximately 50% of all 375 Z-linked genes, highlighting its role in mediating Z chromosome dosage compensation.

Thanks for this comment, this has been added (including the reference to the now published paper):

> *… Theoretical models centered on sexual selection differences between the Z and X chromosomes [7], along with transcriptome analysis in the "model" ZW organism, the chicken, initially led to the assumption that DC would be entirely absent in ZW systems [8] or, if present, would act only on few dosage-sensitive genes [9]. Recent evidence, however, suggests that chicken DC is achieved at posttranscriptional levels [10] via a molecular mechanism involving a male-essential miRNA [11]. In addition, lepidopteran …*

Lines 52-4 – “While mammals and C.elegans use repressive chromatin modifications in XX individuals,”

This represents only the second step in the dosage compensation mechanism. It is important to mention that a first step involves the upregulation of X chromosome activity to balance gene expression from the single X chromosome in males with that of the two sets of autosomes. This phenomenon of X-chromosome upregulation has been well documented in recent studies, including work in C. elegans (DOI: 10.1093/gbe/evaf061) and in certain eutherian mammals (DOI: 10.1126/science.abd8887).

This is a fair point and we agree that upregulation of the mammalian X compared to autosomes is a complex process involving transcriptional and posttranscriptional layers (see e.g. (Rücklé et al., 2023)). Since our paper focuses on ZW species, we had decided to not extensively discuss these aspects. Nevertheless we adjusted the phrasing in the introduction as suggested to also reflect the upregulation:

> *In the well characterized XY model systems (mammals, the nematode Caenorhabditis elegans, the dipteran Drosophila melanogaster) different molecular mechanisms are found for DC [17]. Mammals and C. elegans use repressive chromatin modifications in XX individuals along with compensatory mechanisms that restore X-to-Autosome balance [18–20]. Male fruit flies upregulate the single X in order to match the expression to the XX females [21]. This is achieved via H4K16ac, deposited by the Male-specific lethal (MSL) complex, which …*

Fig. 2C – The figure legend incorrectly states "male (left) and female (right)"; this should be corrected to "female (left) and male (right)."

Please apologize, this has been corrected.

Line 173 – Paragraph: Absence of sex-specific patterns of MOF-MSL complex in *Artemia*.

The data presented are clear and compelling; however, some interpretations appear somewhat overstated. The absence of sex-specific transcription does not rule out other layers of sex-specific regulation. For instance, in *Drosophila*, Msl-2 protein is expressed exclusively in XY cells due to post-transcriptional regulation, including sex-specific splicing and translational repression in the cytoplasm. Therefore, a sex-specific pattern of MOF activity in *Artemia* could still occur in the absence of transcriptional sex bias. Some of the claims should remain framed as hypotheses—for example, line 240: “This provides further support for the idea that H4K16ac is deposited by an MSL-independent mechanism” should be presented more cautiously.

The reviewer is correct that MSL2 protein expression is controlled at posttranscriptional levels, but we would like to draw the reviewers’ attention to the fact that *differential expression* of the *msl-2* (and *msl-3/mof*) transcript between fruit fly males and females can - using sensitive state-of-the-art techniques like RNA-seq - already be observed at steady-state RNA levels (see e.g. **Fig. S3B**).

We have clarified the text as follows:

> … Accordingly, three (*msl-2*, *msl-3* and *mof*) out of four core MSL complex subunits show significantly male-biased expression (Fig. S3B) *(Mean normalized read counts in males (M) and females (F): msl-2 F 408, M 1645; msl-3 F 448, M 821; mof F 311, M 1084).* Like H4K16ac, MSL proteins localize to a male-specific territory …

We see no such sex-specific pattern in *Artemia* like the very obvious one of *Drosophila*. We now also added the suggested splicing analysis (new **Fig. S3C**), which show that none of the MSL orthologues in *Artemia* show sex-linked splicing differences like in *Drosophila*.

Besides looking at *msl* genes, we also ran intron-retention finder (IRFinder) as it was successful in previous work from our group, where we uncovered a novel DC regulator in *A. gambiae* (Kalita et al., 2025, 2023)). Employing a this approach in our embryo dataset revealed in total 5 significant (*padj*<0.05) sex-biased intron retention events, of which all, after manual inspection appeared false positive due to misannotation of the underlying transcript (new **S4 Data**)

In addition, we also performed a new analysis regarding a putative lncRNA alike *roX1/2* (see answer to next point).

Although these new data support our claims, they of course do not exclude MSL involvement *a priori.* We thus also went through the manuscript to ensure there are no overstatements with regards to these - in our view - very interesting findings.

Along the same lines, the authors should expand the discussion of their transcriptional profiling results, particularly regarding any protein-coding genes or non-coding RNAs with sex-specific expression. For instance, what is the expression status of factors from the Human antigen R (HuR/RRM) family? This is relevant given that the Vicoso lab recently identified an AT-rich motif highly enriched in the S0 region of the Z chromosome that may be involved in targeting the dosage compensation machinery. This motif was previously characterized as a binding site for HuR proteins (https://doi.org/10.1093/molbev/msaf085).

We thank the reviewer for these questions. Regarding the HuR proteins, we have added their expression profiles as requested (new **Fig. S3B**, right panel) none of them show a female-biased pattern.

Of course it remains possible that another protein binds to this motif, or that another component of a putative HuR-DC-complex makes the *Artemia* mechanism female/Z-specific. Or HuR is controlled at protein levels? Another possibility is that this motif is enriched on Z by coincidence but is not the guiding principle underlying *Artemia* DC. Elucidating all of this would certainly be exciting, though we hope the reviewer agrees that it represents a distinct project to be explored in future work.

Regarding the question about non-coding RNAs, we have now used the approach by (Tenorio et al., 2024) to identify putative non-coding RNAs expressed from the Z(diff) or PAR functioning like *Xist* / *roX1/2 (*new **Fig. S5H***)*. In brief, this approach is based on binned genome

intervals (50bp) to search for sex-biased transcripts. It has the advantage that it does not rely on previous annotation - and thus - can identify any region showing sex-biased transcripts. This approach has been highly successful in the non-model organism *A. caroliensis* to identify the lncRNA *MAYEX* involved in DC (Tenorio et al., 2024). Performing this analysis in our embryo data, where such a lncRNA should be expressed along with the onset of DC, we could not find any relevant hits for the Z chromosome. This new analysis has been added to the manuscript.

As for the request for further analysis of the transcriptome data: There are 290 genes with higher, and 473 with lower expression in females compared to males. That is by far too many genes, especially given the uncertainty in gene annotation in this non-model organism to come up with any meaningful DC candidate that we could discuss or potentially validate.

Fig. 4 and Fig. 5 – The figure labels appear to be inverted and should be corrected to match the appropriate content; what is currently labeled as Fig. 4 corresponds to Fig. 5, and vice versa.

Please apologize, we have corrected this in the revised manuscript.

Fig. 4C – The legend states that the three replicates are shown as overlays with different shades, but these are not visible in the figure.

We apologize for this - we have now added the previously missing color legend (revised **Fig. 5E**) for the two panels (we also played with different color choices, but unfortunately, this did not improve the clarity further). To see the differences between the replicates better, we have now added a principal component analysis (**Fig. S6B**), which is an independent visualization method to see variability / differences between samples.

Line 257 – "In old females, H4K16ac levels on the female Z(diff) became more variable.", and Line 263 – "The increased variability in H4K16ac was accompanied by gene expression changes".

I find the proposed model difficult to interpret. H4K16ac appears to become slightly more variable specifically on the Z(diff), yet the observed changes in gene expression are not restricted to this region. Moreover, all Z(diff) genes examined show a slight increase in expression, which is counterintuitive given that a decrease in H4K16ac would typically be expected to reduce gene expression. As previously mentioned, the chosen time point is extremely late in the lifespan. Based on the presented data—particularly the lack of significant changes in the acetylation profile—I would draw the opposite conclusion from the authors: that dosage compensation remains stable and is not notably affected by aging.

Ageing is a complex, organismal phenomenon that cannot be caused by a single genomic region (Z(diff)), which we certainly did not intend to claim. What we see is likely a combination of primary and secondary effects - which will affect autosomes and sex chromosomes.

The reviewer mentions a "decrease in H4K16ac" on Z(diff), but this is a misunderstanding, as Z(diff) H4K16ac remains the same (**Fig. S6A**). The reviewer is right that the qPCR gives the

impression that the levels increase and while we acknowledge that there might be a trend, it is not statistically significant. The direction of the change may additionally be influenced by other acetylation marks changing upon ageing that we have not measured.

The point of this figure was to show that like H4K16ac, that gene expression levels can become more variable between individuals, without implying direct causation. We hope that the reviewer is satisfied with the improved clarity / phrasing at several instances in the text and discussion.

Line 280 - "Altogether, our findings are consistent with the idea of inappropriate DC becoming a vulnerability when heterogametic individuals get older. Accordingly, perturbations in H4K16ac would affect the X-linked genes in *Drosophila* males, while they impact Z-linked genes in *Artemia* females."

The authors should revise this paragraph and temper their interpretation to more accurately reflect the data. There is no direct evidence supporting this claim, and the data appear to suggest the opposite. PAR gene expression becomes also more variable with age (Fig. 4E), and nicotinamide treatment affects gene expression more broadly on the PAR and autosomes than on the Z(diff) region in females (Fig. 5D). In addition, H4K16ac levels on the Z chromosome do not show significant age-related changes (Fig. 4C). Taken together, these findings do not support the idea that dosage compensation of the Z chromosome creates a specific vulnerability in aged individuals. On the contrary, the data suggest that other genomic regions—particularly the PAR and autosomes—are more susceptible to aging-associated changes.

We revised this paragraph, but would like to emphasize that:

The H4K16ac ***variability*** changes are substantial and statistically significant:

> *… Interestingly, between old individuals the H4K16ac levels on the female Z(diff) became more variable, as indicated by a significant increase (p<2e-16) in the coefficient of variation (CV, standard deviation divided by the mean CUT&Tag signal per gene across individuals) (Fig. 5F), which was independently confirmed with a principal component analysis (Fig. S6B). Analysis of the CV showed that this effect was specific to the female Z(diff): neither female autosomes nor Z-PAR regions, nor any region …*

Related to the argument that nicotinamide also affects PAR and autosomes - this is not unexpected as any treatment / mutation will lead to a combination of primary and secondary effects on gene expression. The same is true also for the *Drosophila msl*-mutants (see following Figure from (Valsecchi et al., 2020)) - gene expression changes are not only observed on X, but are also substantial on autosomes:

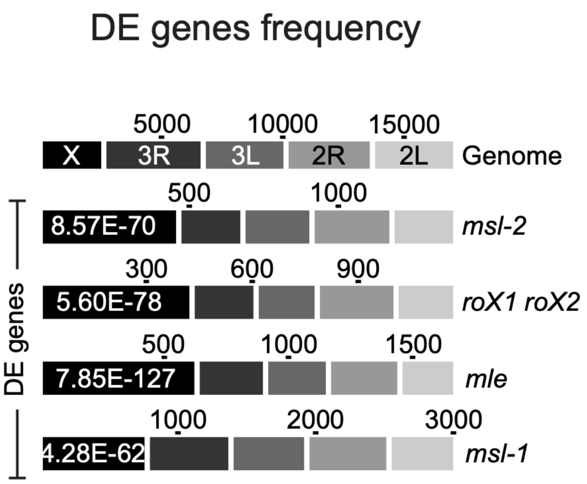

Reviewer Figure 3 - taken from (Valsecchi et al., 2020): Number of differentially expressed genes on X (black) and autosomes in RNA-seq from different *Drosophila* DC mutants (grey shaded bars)).

Along the same lines, it is also not unexpected that PAR genes become more variable, since ageing is a complex phenomenon that cannot be linked to a single genomic region. There are also many more genes residing on autosomes (*n*=13954) and PAR (*n*=741) compared to Z(diff) (*n*=242), so simply by chance the likelihood for a gene expression change on autosomes is highest.

We hope that the reviewer is satisfied with the amendments in the text, along with removal of the nicotinamide experiments.

Line 293 – "This revealed that several factors change expression but also show increased variability in old females, likely perturbing histone acetylation homeostasis."

Why would gene expression variability be higher in older females? Could the authors clarify whether the tested genes are Z-linked? If these effects were due to a degradation of dosage compensation, the findings appear counterintuitive—since a decrease in H4K16ac is generally associated with reduced, not increased, gene expression. Furthermore, the magnitude of the observed changes is relatively small, which raises questions about their potential biological relevance.

The tested genes are autosomal and this has been added (revised **Fig. 6B**). We do not show a decrease / degradation of H4K16ac, rather we show that the signal becomes more ***variable*** specifically on the Z (diff). We then argue that the expression of these genes encoding histone acetylation writers and erasers becomes variable overall in old females and thus, *could* be the upstream effects responsible for H4K16c variability.

We fully agree with the reviewer, whose comments reflect a deep expertise in DC, that even relatively small differences in dosage can be biologically meaningful. Although the changes (**Fig. 6B**) are modest, they are statistically significant for *SIRT1*, which shows a ~ 1.5-fold increase (p = 0.046). Given the reviewer's deep understanding of DC, we trust that they, like us, appreciate that such dosage differences - while perhaps small in magnitude - can have important biological consequences.

However, to test the biological significance of these regulators in old females, we would need genetic tools (i.e. knock-out or knock-down, degrons for specific perturbation in old females), which are unavailable for *A. franciscana*.

Line 307-9 – “Interestingly, the sex-specific differences in lifespan observed under control conditions were alleviated in nicotinamide, indicating that the susceptibility to SIRT1 inhibition differs between males and females.”

This sentence needs clarification, as it is quite misleading. The effect of nicotinamide treatment on longevity is extremely strong and appears more consistent with toxicity than with regulated lifespan modulation. In males, the median adult lifespan drops from approximately 18 days to less than 5 days—a reduction of about 75%. Moreover, all individuals, regardless of sex, die rapidly. At this concentration, nicotinamide is clearly toxic and lethal to *Artemia*.

Please see our previous reply and **Reviewer Fig. 2** - although the nicotinamide experiment was removed from the current paper, there is no toxicity.

Line 310 – “To elucidate how the nicotinamide treatment alters gene expression, we performed RNA-seq from n=4 adult age-matched males and females subjected to nicotinamide for 30 days from hatching onwards in comparison with untreated controls.”

The rationale for selecting a 30-day treatment time point is unclear and should be explicitly justified. Given that the median adult lifespan under nicotinamide treatment is approximately 5 days (Fig. 5C), sampling at 30 days raises concerns that the data may reflect dying or already dead animals, rather than specific biological responses.

We appreciate the reviewer’s careful analysis of our nicotinamide data (now removed from the manuscript). Nevertheless, we would like to answer the question and refer to the new lifespan experiment we performed in the presence and absence of nicotinamide using a slightly refined setup and an increased number of replicates (see above; **Reviewer Fig. 2**). At day 5 of adulthood (30 days of total lifespan), over half of the males are still alive. The individuals we sampled in RNA-seq were certainly not dead; they were actively moving and showed no visible morphological or physiological abnormalities. These animals had been dissected prior to RNA-seq (for removal of gonads), during which any such issues would have been apparent.

Nevertheless, we have also examined the expression of apoptosis-related marker genes in our RNA-seq dataset (**Reviewer Fig. 4**): DrICE (caspase-3–like), API5, and HtrA2/omi. DrICE, an effector caspase, whose expression is expected to increase in apoptotic cells, was in fact slightly higher in controls compared to nicotinamide-treated individuals. API5 and HtrA2/omi showed no major differences between the two groups. These results do not indicate an increase in apoptotic activity in the nicotinamide-treated animals, supporting our conclusion that the sampled individuals were not in the process of dying.

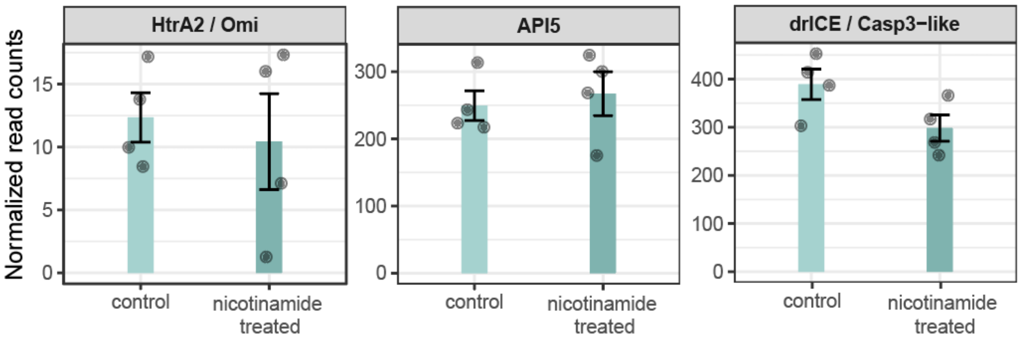

Reviewer Figure 4: RNA levels (Normalized read counts from RNA-seq) in Nicotinamide-treated in comparison with control *Artemia* for selected genes related to cell death.

Line 378 – "By revealing a link between misregulated DC and ageing, our data can further provide one mechanistic explanation for reduced longevity of the heterogametic sex in both XY and ZW species."

These are overstatements that should be corrected. H4K16ac levels on the Z chromosome do not show significant age-related changes, and the findings presented are too preliminary to support the claim that Z chromosome dosage compensation creates a specific vulnerability in aged individuals.

This paragraph has been rephrased.

Line 383 – "Unexpectedly, roX1 lncRNA has been recently uncovered as a fruit fly ageing biomarker and interfering with roX1 function partially alleviates sex-specific differences in lifespan [56]. Although the effect of MSL-H4K16ac in the *Drosophila* context (roX1 mutant) remains to be investigated, one possibility is a link to DC."

Not exactly. The rox1[SMC17A] mutant specifically affects female lifespan and has no impact in males, where MSL-mediated dosage compensation occurs. By definition, the novel function of rox1 described here is therefore independent of the dosage compensation pathway.

The reviewer is correct that the *roX1* effect on ageing occurs in females and thus, this reference does not support a link between MSL-H4K16ac and ageing. Thus, we have removed these sentences

Line 390 – "Mechanistically, this could be linked to mechanisms like epigenetic noise and transcriptional instability [74], observed by us here in *Artemia* but also found in other systems like mammals. Increased cell to-cell transcriptional variability occurs for example in the ageing mammalian immune system [75]. In mammals, genes that escape X inactivation - resulting in biallelic expression in XX females but single-copy expression in XY males - may play a crucial role that is worthwhile investigating in the future [60,76]."

The proposed model is unclear. If aging in XX females were driven by erosion of X inactivation leading to biallelic expression, one would expect this to result in reduced lifespan. However, XX females generally live longer than males. If dosage compensation indeed created a

vulnerability in mammals, this pattern would be reversed. The model, as currently presented, does not align with known sex differences in lifespan.

During the revision, two studies were published reporting X chromosome reactivation in ageing mammals (Gadek et al., 2025; Hoelzl et al., 2025), supporting a model in which erosion and context-dependent biallelic expression of X-linked genes may confer protective effects in females. Conversely, other findings suggest that age-associated escape from X inactivation - such as increased expression of *Tlr8*, an X-linked gene known to escape in immune cells - can contribute to autoimmune conditions like late-onset lupus (Adeogun et al., 2024). Of note, Xist silencing has been shown to depend on anchoring to the nuclear lamina (Chen et al., 2016), a structure recognized as a key player in the ageing process. Therefore, whether erosion of DC is beneficial or deleterious likely depends on cellular context and the specific genes involved - which remains an active and exciting area of investigation. We find it particularly intriguing to observe a conceptually similar phenomenon in *Artemia*, suggesting this process may be evolutionarily conserved. We have revised our phrasing accordingly and hope the link between dosage compensation and ageing is now more clearly articulated.

> … sex-specific ageing processes. Mechanistically, this could be linked to mechanisms like epigenetic noise and transcriptional instability [79], observed by us here in *Artemia* but also found in other systems like mammals (e.g. in the immune system [80]). *In mammals, loss of DC upon ageing has been linked to both beneficial and deleterious outcomes. Age-related escape from X inactivation can lead to biallelic expression in XX females - versus single-copy expression in XY males - and has been associated with improved cognitive function and protection against pulmonary fibrosis [81–84]. Conversely, the age-associated escape of Tlr8 could promote autoimmune conditions like late-onset lupus [85].* This raises the possibility that while DC is critical early in life for correcting dosage imbalances, its modulation later in life could be beneficial, but could also create an "epigenetic vulnerability" affecting sex-specific traits and disease risk.

Title – "Convergent Evolution of H4K16ac-mediated Dosage Compensation Shapes Sex-dependent Lifespan in a ZW Species."

The second part of the title, "shapes sex-dependent lifespan in a ZW species", should be removed or significantly revised, as the current data do not provide sufficient evidence to support such a strong and causal claim.

Abstract – The following three sentences should be substantially revised to more accurately reflect the data:

-"H4K16ac-mediated DC is established during embryogenesis, sustained until adulthood, but then becomes reconfigured and variable in aged females."

This statement is not supported by the data, as the authors themselves report no significant age-related changes in H4K16ac levels on the Z chromosome (Fig. 4C).

-“Interfering with acetylation alleviates male-female differences in lifespan.”

This is misleading. The effect of nicotinamide treatment is extremely severe and suggests general toxicity rather than modulation of sex-specific lifespan. In males, the median lifespan drops from ~18 days to <5 days—a ~75% reduction—and both sexes die rapidly. At the tested concentration, nicotinamide appears to be broadly toxic and lethal to *Artemia*, not a targeted regulator of sex-specific longevity.

-“They highlight histone acetylation as a sex-specific vulnerability linked to the chromatin landscape of sex chromosomes and its potential role in driving male-female differences.”

This claim overstates the findings. The presented data are preliminary and do not support the conclusion that dosage compensation of the Z chromosome constitutes a sex-specific epigenetic vulnerability in aging.

We have amended title and abstract substantially taking into consideration these valid points raised by the reviewer. The connection of H4K16ac to ageing is now kept minimal and phrased as a hypothesis.

Reviewer #3

This is a very nice contribution to our understanding of the evolution of dosage compensation. The conclusions are mostly well supported. In particular, the data presented in Figure 2 and 3 especially. I have some minor comments, but I do have one major concern that should be addressed either with new experiments or a very clear statement hedging their conclusions.

Major point: One conclusion from figure 1 might be that Plodia, Bacillus, Anopheles, Leptinofarsa and Reticulitermes and Triops do not achieve dosage compensation on the Z or W in the heterogametic sex through H4K16ac. However, that specific conclusion is not supported because the IF they do doesn't provide information about the sex chromosomes. The sex chromosome may dispersed within the nuclei, still significantly marked with H4K16ac, but simply not partitioned in a distinct territory. The specific conclusion that can be made is that there is no distinct H4K16ac TERRITORY in the heterogametic sex. To conclude that there is no DC by way of H4K16ac in the heterogametic sex, they would have to either mark the sex chromosome with FISH, or do chromatin profiling with ChIP seq or cut and run. As such, they must provide a clear statement indicating that they may only conclude that any possible dosage compensation in these species may still occur through H4K16ac, but that there doesn't appear to be a paritioning as seen in *Drosophila*, Bradysia and *Artemia*.

For revision, we have added genome-wide profiling data for two species without territory (see also reply to Reviewer #1): *Bacillus grandi* (new CUT&Tag data) and *Anopheles gambiae* (ChIP-seq, from (Keller Valsecchi et al., 2021)). Those confirm the immunofluorescence findings: There is no sex-specific enrichment of H4K16ac on the X chromosome and thus, this mark does not contribute to DC as in *Drosophila* or *Artemia*. We would like to note that immunofluorescence is an attractive approach, when no high-quality genome assemblies are available (e.g. in the termite *R. flavipes*). Besides this new data (**Fig. 2A-B, Fig. S2A-B**), we have ensured that our manuscript does not contain overstatements in that regard.

Minor points:

Line 50: Italicize *A. franciscana*

Fixed.

Line 172: Worth mentioning the RNA-seq result for 1F which supports their conclusion. This doesn't appear mentioned in the text.

This has been added.

How is FTZ dynamically expressed in S5C? I don't see it. Likewise for nAChRB1. Others, perhaps so. Please be more upfront about this. It is hard to know, with the variation presented, if there is some significant difference across stages.

Our phrasing has been indeed imprecise - we changed it to:

time-points clustered separately (Fig. S5A) and differential gene expression analysis identified 739 up- and 445 downregulated genes (4 dpf vs. 1-2 dpf; Fig. S5B). In addition, we examined the expression of Artemia orthologues of key early developmental regulatory genes in Drosophila. Observing that the pair-rule gene fushi tarazu (FTZ), head gap gene empty spiracles (EMX2), Nicotinic Acetylcholine Receptor Subunit b1 (nAChRb1, nervous system development) and the transcription factor PAX5 (midbrain development) are expressed at the 1-2 dpf time-point indicates that embryos had passed zygotic genome activation, while their expression dynamics confirms that the 4 dpf embryos undergo tissue specification and organogenesis (post-gastrulation) (Fig. S5C). RPL19 - a housekeeping gene - appeared stable, while the homeobox gene UBX - whose function may have diverged in Crustacea [60] - was not expressed.

Are 4 and 5 switched with respect to order???

Please apologize, we have corrected the mistakes with regards to Figure labelling and callouts in the revised manuscript.

Provide some visual that demonstrates source of variation in 4D. Is this always driven by a single sample? Or, is it variable across samples (ie, in one sample, one gene in z(diff) is quite different from the other two, but, in the same sample comparison, another gene shows a different pattern. Could this be driven by a single old sample? This needs to be clarified.

To analyze this, we have added a principal component analysis (new **Fig. S6B**), in which the distance between data points (=individuals) reflects the similarity or dissimilarity between replicates, with closer points indicating greater similarity and vice versa. From this, it can be seen that all three samples cluster differently from each other, meaning, the variation is not driven by a single sample (otherwise, two samples would cluster very close, and one clustering separately).

Overall, I enjoyed this paper and I think it is a nice contribution.

We thank the reviewer for their positive evaluation of our manuscript.

References

- Adeogun G, Camai A, Suh A, Wheless L, Barnado A. Comparison of late-onset and non-late-onset systemic lupus erythematosus individuals in a real-world electronic health record cohort. Lupus 2024;33:525–31.
- Bett VK, Trejo Arellano MS, Vicoso B. Chromatin Landscape is Associated with Sex-biased Expression and *Drosophila*-like Dosage Compensation in *Artemia franciscana*. Mol Biol Evol 2025:msaf085.
- Chen C-K, Blanco M, Jackson C, Aznauryan E, Ollikainen N, Surka C, et al. Xist recruits the X chromosome to the nuclear lamina to enable chromosome-wide silencing. Science 2016;354:468–72.
- Fritz García JHG, Keller Valsecchi CI, Basilicata MF. Sex as a biological variable in ageing: insights and perspectives on the molecular and cellular hallmarks. Open Biol 2024;14:240177.
- Gadek M, Shaw CK, Abdulai-Saiku S, Saloner R, Marino F, Wang D, et al. Aging activates escape of the silent X chromosome in the female mouse hippocampus. Sci Adv 2025;11:eads8169.
- Hägg S, Jylhävä J. Sex differences in biological aging with a focus on human studies. Elife 2021;10. https://doi.org/10.7554/eLife.63425.
- Hoelzl S, Hasenbein TP, Engelhardt S, Andergassen D. Aging promotes reactivation of the Barr body at distal chromosome regions. Nat Aging 2025;5:984–96.
- Kalita AI, Marois E, Kozielska M, Weissing FJ, Jaouen E, Möckel MM, et al. The sex-specific factor SOA controls dosage compensation in Anopheles mosquitoes. Nature 2023;623:175–82.
- Kalita AI, Marois E, Rühle F, Keller Valsecchi CI. Sex-specific transcriptome dynamics of Anopheles gambiae during embryonic development. Genes Dev 2025. https://doi.org/10.1101/gad.352572.124.
- Keller Valsecchi CI, Marois E, Basilicata M, Georgiev P, Akhtar A. Distinct mechanisms mediate X chromosome dosage compensation in Anopheles and *Drosophila*. Life Sci Alliance 2021;4. https://doi.org/10.26508/lsa.202000996.
- Rücklé C, Körtel N, Basilicata MF, Busch A, Zhou Y, Hoch-Kraft P, et al. RNA stability controlled by m6A methylation contributes to X-to-autosome dosage compensation in mammals. Nat Struct Mol Biol 2023. https://doi.org/10.1038/s41594-023-00997-7.
- Tenorio M, Cruz-Ruiz S, Encarnación-Guevara S, Hernández M, Corona-Gomez JA, Sheccid-Santiago F, et al. MAYEX is an old long noncoding RNA recruited for X chromosome dosage compensation in a reptile. Science 2024;385:1347–54.
- Valsecchi CIK, Basilicata M, Georgiev P, Gaub A, Seyfferth J, Kulkarni T, et al. RNA nucleation by MSL2 induces selective X chromosome compartmentalization. Nature 2020;589:137–42.

- Adeogun G, Camai A, Suh A, Wheless L, Barnado A. Comparison of late-onset and non-late-onset systemic lupus erythematosus individuals in a real-world electronic health record cohort. Lupus 2024;33:525–31.
- Bett VK, Trejo Arellano MS, Vicoso B. Chromatin Landscape is Associated with Sex-biased Expression and *Drosophila*-like Dosage Compensation in *Artemia franciscana*. Mol Biol Evol 2025:msaf085.
- Chen C-K, Blanco M, Jackson C, Aznauryan E, Ollikainen N, Surka C, et al. Xist

recruits the X chromosome to the nuclear lamina to enable chromosome-wide silencing. Science 2016;354:468–72.
- Fritz García JHG, Keller Valsecchi CI, Basilicata MF. Sex as a biological variable in ageing: insights and perspectives on the molecular and cellular hallmarks. Open Biol 2024;14:240177.
- Gadek M, Shaw CK, Abdulai-Saiku S, Saloner R, Marino F, Wang D, et al. Aging activates escape of the silent X chromosome in the female mouse hippocampus. Sci Adv 2025;11:eads8169.
- Hägg S, Jylhävä J. Sex differences in biological aging with a focus on human studies. Elife 2021;10. https://doi.org/10.7554/eLife.63425.
- Hoelzl S, Hasenbein TP, Engelhardt S, Andergassen D. Aging promotes reactivation of the Barr body at distal chromosome regions. Nat Aging 2025;5:984–96.
- Kalita AI, Marois E, Kozielska M, Weissing FJ, Jaouen E, Möckel MM, et al. The sex-specific factor SOA controls dosage compensation in Anopheles mosquitoes. Nature 2023;623:175–82.
- Kalita AI, Marois E, Rühle F, Keller Valsecchi CI. Sex-specific transcriptome dynamics of Anopheles gambiae during embryonic development. Genes Dev 2025. https://doi.org/10.1101/gad.352572.124.
- Keller Valsecchi CI, Marois E, Basilicata M, Georgiev P, Akhtar A. Distinct mechanisms mediate X chromosome dosage compensation in Anopheles and *Drosophila*. Life Sci Alliance 2021;4. https://doi.org/10.26508/lsa.202000996.
- Rücklé C, Körtel N, Basilicata MF, Busch A, Zhou Y, Hoch-Kraft P, et al. RNA stability controlled by m6A methylation contributes to X-to-autosome dosage compensation in mammals. Nat Struct Mol Biol 2023. https://doi.org/10.1038/s41594-023-00997-7.
- Tenorio M, Cruz-Ruiz S, Encarnación-Guevara S, Hernández M, Corona-Gomez JA, Sheccid-Santiago F, et al. MAYEX is an old long noncoding RNA recruited for X chromosome dosage compensation in a reptile. Science 2024;385:1347–54.
- Valsecchi CIK, Basilicata M, Georgiev P, Gaub A, Seyfferth J, Kulkarni T, et al. RNA nucleation by MSL2 induces selective X chromosome compartmentalization. Nature 2020;589:137–42.
